# Inhibition of cyclin‐dependent kinases 12/13 using CT7439 as a treatment for colorectal cancer with CDK12 upregulation

**DOI:** 10.1002/1878-0261.70290

**Published:** 2026-06-30

**Authors:** Wylie K. Watlington, Divya L. Dayanidhi, Mohammad Zokaasadi, John B. Mantyh, Pelumi D. Olawuni, Gabrielle Rupprecht, Jeremy M. Force, Shannon McCall, Ashwani K. Bahl, David S. Hsu, Jason A. Somarelli

**Affiliations:** ^1^ Department of Medicine, Division of Medical Oncology Duke University Medical Center Durham NC USA; ^2^ Center for Genomics and Computational Biology Duke University Durham NC USA; ^3^ Daiichi Sankyo, Inc. Basking Ridge NJ USA; ^4^ Department of Pathology Duke University Durham NC USA; ^5^ Carrick Therapeutics, Inc. Dublin Ireland

**Keywords:** CDK12, CDK12/13 inhibition, CDK13, colorectal cancer, patient‐derived organoids

## Abstract

Cyclin‐dependent kinase (CDK) 12 and its paralog, CDK13, phosphorylate RNA polymerase II, enabling transcriptional elongation. In solid tumors, CDK12 loss promotes progression by inducing replication‐transcription conflict and fueling genomic instability. However, we have uncovered upregulation of CDK12 and CDK13 in ~5% of colorectal cancer (CRC) specimens, suggesting a role in cancer cell survival. Based on this, we postulated that CDK12/13 inhibition in CRC may represent a useful therapeutic strategy. To test this, we screened CDK12 and CDK12/13 inhibitors across multiple cancer cell lines and patient‐derived organoids (PDO) from a range of solid tumors, demonstrating potent activity in CRC PDO. Using siRNA‐mediated knockdown, we identified CDK13 as a potential mechanism of resistance to CDK12‐specific inhibition. Mechanistically, CDK12/13 inhibition led to a decreased abundance of BRCA1 long transcripts, rendering cells susceptible to combination therapy with PARP inhibitors. To further assess the clinical utility of CDK12/13 inhibition, we focused on CRC, for which there is an urgent need for additional therapies. We tested the efficacy of CT7439, a novel CDK12/13 inhibitor and cyclin K degrader, which showed cytotoxicity in the low nanomolar range, reduced BRCA1 expression, and concomitant DNA damage. Together, our data support further clinical development of CDK12/13 inhibition in CRC.

## Graphical abstract

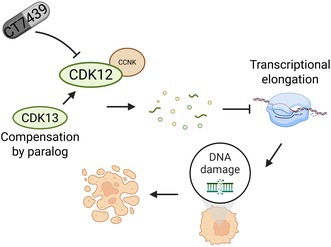

**Abbreviations**
BRPC: BioRepository and Precision Pathology Center
CDK: cyclin‐dependent kinase
CRC: colorectal cancer
CTD: c‐terminal domain
H&E: hematoxylin and eosin
PARP: poly‐ADP ribose polymerase
PDO: patient‐derived organoid
pol: polymerase
PROTAC: proteolysis‐targeting chimera
SD: standard deviation

## 1 Introduction

A major hallmark of tumorigenesis is uncontrolled cell proliferation [1]. The regulation of proliferation is mediated by numerous cell‐extrinsic signals, including cell‐matrix attachment [2], ligand‐receptor interactions [3], and nutrient sensing [4]. These signals modulate cell cycle progression by altering the expression and/or activity of a series of cell cycle regulators [5]. Cell cycle regulatory proteins include cyclins and cyclin‐dependent kinases (CDKs), which typically promote cell cycle progression, along with two families of cell cycle inhibitory proteins, the INK4 family (p14^ARF^, p15^INK4b^, p16^INK4a^, p18^INK4C^, p19^INK4D^) and the Cip/Kip family (p21^Cip1^, p27^Kip1^, p57^Kip2^) [6].

In addition to direct regulation of the cell cycle, regulation of transcription and translation also impact proliferative capacity, and several members of the CDK family modulate transcription and post‐transcriptional processing [7, 8]. Many transcriptional CDKs catalyze phosphorylation of the c‐terminal domain (CTD) of RNA polymerase (pol) II, regulating its activity [9]. CDK12, along with its corresponding cyclin, cyclin K, functions by phosphorylating serine 2 of a repeating heptapeptide sequence in the RNA pol II CTD [10]. The CTD of RNA pol II helps organize phases of transcription through regulated protein binding, thus making it a necessary function for the cell [11]. Phosphorylation of the RNA pol II c‐terminal domain by CDK12 regulates transcription elongation, particularly for long mRNAs. Biallelic loss of CDK12 in cancer cells can result in increased DNA damage, as CDK12 loss disrupts expression of multiple long transcripts involved in the DNA damage response pathway, such as BRCA1 [12]. The increased DNA damage upon loss of CDK12 renders cancer cells susceptible to poly‐ADP ribose polymerase (PARP) inhibition [13, 14, 15].

While much of the focus on CDK12 in cancer has been on genomic loss, CDK12 can also be upregulated and play a role in the progression of cancerous tissues as compared to normal tissues [16]. For example, in papillary thyroid cancer, CDK12 upregulation promotes c‐Myc/β‐catenin activation, and thus is an important component of cancer proliferation [17]. This would render CDK12 an optimal target for therapeutic inhibition, particularly in instances where the protein is upregulated and its inhibition can synergize with PARP inhibition.

In addition to CDK12, CDK13, a closely related paralog of CDK12, has been shown to perform similar roles to CDK12. CDK13 shares 43% amino acid identity and almost 92% identity with CDK12 in the kinase domain. Both CDK12 and CDK13 work by phosphorylating the CTD of RNA pol II; however, despite their similarities in structure, they have also been shown to impact transcriptional elongation of distinct subsets of genes [9]. For example, CDK12 appears to be involved in the DNA damage response, while CDK13 is involved in growth signaling and extracellular processes, suggesting that dual inhibition of CDK12 and CDK13 may have improved efficacy over targeting of CDK12 alone [18].

In this study, we sought to understand the therapeutic potential of CDK12/13 inhibition in solid tumors. To do this, we screened a series of cancer cell lines from breast, colorectal, lung, ovarian, and prostate cancers across a panel of CDK12 and CDK12/13 inhibitors, including covalent and noncovalent binders, molecular glues, and proteolysis‐targeting chimeras (PROTACs). The covalent binders (THZ531 and CDK12‐IN‐E9) were found to be the most efficacious agents across all tumor types including colorectal cancer, and this was further validated in a panel of colorectal cancer (CRC) patient‐derived organoids (PDO). Finally, using siRNA‐mediated knockdown of CDK13 in the context of CDK12‐specific inhibition, we demonstrated that CDK13 can partially compensate for CDK12 inhibition. This suggests that a dual CDK12/13 inhibitor may have an improved benefit, particularly in cancers that exhibit dual upregulation of CDK12 and CDK13. Given the promising results with these compounds, we tested the efficacy of the novel CDK12/13 and cyclin K glue‐degrader CT7439 [19]. CT7439 exhibited potent cytotoxicity in the low nanomolar range across a panel of CRC PDO and synergized with PARP inhibition. Mechanistically, CT7439 led to loss of long isoforms of BRCA1 and resulting DNA damage. Taken together, these observations suggest that inhibition of CDK12/13, and the introduction of CT7439 into the clinical setting, may represent a promising treatment for solid tumors harboring upregulation of CDK12 and CDK13.

## 2 Materials and methods

### 2.1 Analysis of CDK12 and CDK13 alterations in public cancer data sets

Data from breast, colorectal, lung, ovarian, prostate, and other solid tumors were obtained from the Cancer Genome Atlas via cBioPortal and the University of California, Santa Cruz Xena Browser. Default RNA cutoffs of “mRNA expression ≤ 2 standard deviations (SD) below the mean”, “mRNA expression ≥ 2 SD above the mean” were used to query both CDK12 and CDK13. Expression levels of mRNA and genomic alteration data were used to compare expression and alteration of CDK12 and CDK13 and were normalized and presented as a percentage of the total number of samples. The mRNA expression graphs were plotted in GraphPad Prism (La Jolla, CA, USA) (GraphPad Prism, RRID:SCR_002798). Linear regression was performed for each cancer type using the expression levels of CDK12 and CDK13 and plotted using the simple linear regression function in GraphPad Prism (La Jolla, CA, USA) (GraphPad Prism, RRID:SCR_002798). *P*‐values < 0.05 were considered statistically reliable. The 95% confidence intervals for the slope of each data set are as follows: PanCancer [0.36, 0.38]; Prostate [0.44, 0.54]; Ovarian [0.15, 0.37]; Lung [0.28, 0.37]; Colon [0.20, 0.35]; Breast [0.20, 0.25].

### 2.2 Cell line establishment and maintenance

Cell lines from five different cancer types were obtained as fresh frozen viable cells from the Duke University Cell Culture Facility. The cell lines in this panel were RKO (RRID:CVCL_0504), HCT‐15 (RRID:CVCL_0292), HT‐29 (RRID:CVCL_0320), LNCaP (RRID:CVCL_0395), DU‐145 (RRID:CVCL_0105), PC‐3 (RRID:CVCL_0035), AU565 (RRID:CVCL_1074), HCC2218 (RRID:CVCL_1263), ZR‐75‐1 (RRID:CVCL_0588), SUM149PT (RRID:CVCL_3422), HCC1937 (RRID:CVCL_0290), T‐47D (RRID:CVCL_0553), MCF‐7 (RRID:CVCL_0031), HCC1954 (RRID:CVCL_1259), HCC38 (RRID:CVCL_1267), NCI‐H23 (RRID:CVCL_1547), NCI‐H460 (RRID:CVCL_0459), A549 (RRID:CVCL_0023), NIH:OVCAR‐3 (RRID:CVCL_0465), and SK‐OV‐3 SK‐OV‐3 (RRID:CVCL_0532). All cell lines were authenticated using STR profiling through the Duke Life Science Facility, and all experiments were performed with mycoplasma‐free cells. The low passage patient‐derived colorectal cancer cell line CRC cell line 1 was developed from a patient‐derived tumor. The low passage patient‐derived colorectal cancer cell line CRC cell line 2 was developed from a patient‐derived xenograft [20]. All experiments were performed with mycoplasma‐free cells. All frozen vials of these cell lines were thawed in a 37 °C water bath, and the freezing media was quenched with 10 mL DMEM F12 with 10% FBS and 1% penicillin/streptomycin. The solution was centrifuged at 750× ***g*** for five minutes, and supernatant was aspirated. A total of 1 × 10^6^ cells were plated in either a 10 cm cell culture dish (for adherent lines) or a T75 flask (for suspension lines) in 10 mL of culture media and 1% penicillin/streptomycin. CRC cell line 1 and 2 cells were cultured in DMEM F12 with 10% FBS and 1% penicillin/streptomycin. All cell lines were maintained in a 37 °C incubator with 5% CO_2_.

### 2.3 Establishment and maintenance of patient‐derived organoids

Patients were consented and tissue samples were obtained from the National Cancer Institute's Cooperative Human Tissue Network and the Duke University BioRepository and Precision Pathology Center (BRPC) under a Duke Institutional Review Board–approved protocol (Pro00035974). Experiments were undertaken with the understanding and written consent of each subject, and the study methodologies conformed to the standards set by the Declaration of Helsinki. Duke BRPC performed histological assays on these samples to confirm their status. Samples were then provided to the laboratory of Dr. Hsu under a Duke Institutional Review Board approved protocol (Pro000089222). Tissue samples were cut into pieces ~2 mm^3^ with a sterile scalpel and enzymatically digested in 5 mL tubes with 4.7 mL Advanced DMEM F12 media and manufacturer‐recommended amounts of H, R, and A enzymes in the Tumor Dissociation Kit, human (Miltenyi Biotec). Samples were digested for 60 min at 37 °C in the Roto‐Therm Plus (Ward's Science). Cells and tissue fragments were then filtered through 70 μm filters and centrifuged at 750× ***g*** for five minutes. Supernatants were aspirated, and 2 × 10^5^ cells were plated as PDO in three 50 μL Cultrex® BME, Type 3 (R&D Systems) domes in 24‐well plates. All PDO were maintained in a 37 °C incubator with 5% CO_2_. PDO were considered successfully established after maintaining consistent growth for at least three passages, passaging every ~2 weeks. Most PDO used for this study did not exceed 30 passages.

Breast PDO were grown in Advanced DMEM F12 media supplemented with the following components: 10 mm HEPES, 1× GlutaMax, 100 U·mL^−1^ Penicillin/Streptomycin, 100 nm 17‐B‐Estradiol, 500 nm A83‐01, 1X B27 without Vitamin A, 5 ng·mL^−1^ EGF, 100 pg·mL^−1^ FGF2, 5 ng·mL^−1^ FGF7, 20 ng·mL^−1^ FGF10, 10 μm Forskolin, 0.5 μg·mL^−1^ Hydrocortisone, 1.25 mm N‐Acetylcysteine, 5 nm Neuregulin I, 5 mm Nicotinamide, 100 ng·mL^−1^ Noggin, 100ug·mL^−1^ Primocin, 100 ng·mL^−1^ R‐Spondin 1, 250 ng·mL^−1^ R‐Spondin 3, 500 nm SB202190, and 0.2 nm Wnt‐3a.

Colorectal PDO were grown in Advanced DMEM F12 media supplemented with the following components: 10 mm HEPES, 1× GlutaMax, 500 nm A83‐01, 1× B27 without Vitamin A, 50 ng·mL^−1^ EGF, 50 ng·mL^−1^ FGF2‐G3, 12.5 ng·mL^−1^ FGF7, 20 ng·mL^−1^ FGF10, 100 ng·mL^−1^ IGF‐1, 10 nm [Leu15]‐Gastrin I, 1.25 mm N‐Acetylcysteine, 10 mm Nicotinamide, 100 ng·mL^−1^ Noggin, 100ug·mL^−1^ Primocin, 1 nm Prostaglandin E2, 125 ng·mL^−1^ R‐Spondin 1, and 0.2 nm NGS Wnt.

Lung PDO were grown in Advanced DMEM F12 media supplemented with the following components: 10 mm HEPES, 1× GlutaMax, 100 U·mL^−1^ Penicillin/Streptomycin, 500 nm A83‐01, 1× B27 without Vitamin A, 25 ng·mL^−1^ FGF7, 100 ng·mL^−1^ FGF10, 1.25 mm N‐Acetylcysteine, 5 mm Nicotinamide, 100 ng·mL^−1^ Noggin, 100ug·mL^−1^ Primocin, 500 ng·mL^−1^ R‐Spondin 1, and 500 nm SB202190. Lung PDO 1 was a colon lung met sample, whereas Lung PDO 2–3 were lung primary samples.

Ovarian PDO were grown in Advanced DMEM F12 media supplemented with the following components: 10 mm HEPES, 1× GlutaMax, 100 U·mL^−1^ Penicillin/Streptomycin, 10 nm 17‐B‐Estradiol, 500 nm A83‐01, 1× B27 without Vitamin A, 50 ng·mL^−1^ EGF, 25 ng·mL^−1^ FGF7, 100 ng·mL^−1^ FGF10, 10 nm [Leu15]‐Gastrin I, 10 ng·mL^−1^ HGF, 20 ng·mL^−1^ IGF, 1× N2, 1 mm N‐Acetylcysteine, 10 ng·mL^−1^ Neuregulin I, 10 mm Nicotinamide, 100 ng·mL^−1^ Noggin, 100ug·mL^−1^ Primocin, 10 nm Prostaglandin E2, 100 ng·mL^−1^ R‐Spondin 1, 3 μm SB202190, and 10 μm SB203580 (p38i).

Prostate PDO were grown in Advanced DMEM F12 media supplemented with the following components: 10 mm HEPES, 1× GlutaMax, 100 U·mL^−1^ Penicillin/Streptomycin, 500 nm A83‐01, 1× B27 without Vitamin A, 0.1 nm Dihydrotestosterone, 50 ng·mL^−1^ EGF, 25 ng·mL^−1^ FGF7, 100 ng·mL^−1^ FGF10, 10 nm [Leu15]‐Gastrin I, 10 ng·mL^−1^ HGF, 20 ng·mL^−1^ IGF, 1× N2, 1 mm N‐Acetylcysteine, 10 ng·mL^−1^ Neuregulin I, 10 mm Nicotinamide, 100 ng·mL^−1^ Noggin, 100 ug·mL^−1^ Primocin, 10 nm Prostaglandin E2, 100 ng·mL^−1^ R‐Spondin 1, 3 μm SB202190, and 10 μm SB203580 (p38i).

### 2.4 Screening a CDK12 and CDK12/13 inhibitor panel

Cell lines were allowed to establish until approximately 80% confluent before plating for drug treatments. For adherent cell lines, media was aspirated, and 3 mL of PBS was added to wash the plates. A total of 5 mL of 0.25% Trypsin–EDTA (Gibco) was added, and plates were incubated at 37 °C for 5 min to detach the cells. Following incubation, 5 mL of DMEM F12 Media with 10% FBS and 1% penicillin/streptomycin was added to neutralize the Trypsin reaction. Solutions were centrifuged at 750× ***g*** for five minutes, and supernatants were aspirated. For cell lines in suspension, cells and media were collected from flasks and centrifuged at 750× ***g*** for five minutes. Supernatants were aspirated, and both adherent and suspension cells were plated at 3 × 10^3^ cells per well in 50 μL line‐specific media. The following CDK12 inhibitors were prepared at 10 mm stock solutions in DMSO: THZ531 (covalent), CDK12‐IN‐E9 (covalent), SR4835 (noncovalent), CDK12‐IN‐2 (noncovalent), BSJ‐4‐116 (proteolysis targeting chimera, PROTAC), R‐CR8 (molecular glue), HQ461 (molecular glue), and NCT02 (molecular glue). Using a three‐point dose curve with a dilution factor of 10, the cells were treated with each of the compounds at 50 nm, 500 nm, 5000 nm, or control. Dose response assays were conducted with three replicates per dose. Plates were imaged at 0 and 72 h of treatment using the ImageXpress Pico live cell imaging system. After 72 h, cell viability is estimated using the CellTiter‐Glo Luminescent Cell Viability Assay kit (Promega). Luminescence values were measured using the Varioskan Lux plate reader (Thermo Fisher Scientific). Percent viability was calculated as follows: 100*(average CellTiterGlo_drug_/average CellTiterGlo_control_).

### 2.5 Dose response curves of patient‐derived organoids

PDO were allowed to establish until approximately 80% confluent before plating. Media was aspirated, and 1 mL of PBS was used in each well to detach the 50 μL Cultrex® domes. The solutions were centrifuged at 750× ***g*** for five minutes, followed by the addition of 1 mL of TrypLE Express (Gibco) to dissociate organoids into single cells and dissolve the remaining Cultrex®. After incubating for five minutes, 5 mL of DMEM F12 Media with 10% FBS and 1% penicillin/streptomycin was added to neutralize the TrypLE Express reaction. Solutions were again centrifuged at 750× ***g*** for five minutes, and supernatants were aspirated. Cells were plated in a 96‐well plate at a density of 2 × 10^3^ cells per well in 5 μL Cultrex® domes and treated as follows:

1. CDK inhibition. THZ531, CDK12‐IN‐E9, and CT7439 were prepared at 10 mm stock solutions in DMSO. Using a nine‐point dose curve with a dilution factor of three, the cells were either a control group or treated with THZ531 or CDK12‐IN‐E9 at a starting dose of 50 μm, or CT7439 at a starting dose of 1 μm. Recombinant AlexaFluor® 647 Anti‐EpCAM Antibody (Abcam) was added for THZ531 and CDK12‐IN‐E9‐treated plates at a 1:1000 dilution. Dose response curves were conducted with five technical replicates per dose and one biological replicate per line. Plates were imaged at 0, 24, 48, and 72 h of treatment using the ImageXpress Pico live cell imaging system. After 72 h, cell viability was estimated using the CellTiter‐Glo Luminescent Cell Viability Assay kit (Promega) per the manufacturer's protocol. Luminescence values were measured using the Varioskan Lux plate reader (Thermo Fisher Scientific). Percent viability was calculated as follows: 100*(average CellTiterGlo_drug_/average CellTiterGlo_control_).
2. Standard of Care Chemotherapy. Oxaliplatin was prepared at a 10 mm stock solution in PBS. 5‐Fluorouracil and THZ531 were prepared at 10 mm stock solutions in PBS. SN38 was prepared at a 10 mm stock solution in DMSO. Palbociclib was prepared at a 4 mm stock solution in DMSO. Using a seven‐point dose curve with a dilution factor of 5, the cells were either a control group or treated with each of the compounds at a starting dose of 100 μM. Dose response curves were conducted with five technical replicates per dose and one biological replicate per line. Plates were imaged at 0 and 72 h of treatment using the ImageXpress Pico live cell imaging system. After 72 h, cell viability was estimated using the CellTiter‐Glo Luminescent Cell Viability Assay kit (Promega) per manufacturer protocol. Luminescence values were measured using the Varioskan Lux plate reader (Thermo Fisher Scientific). Percent viability was calculated as follows: 100*(average CellTiterGlo_drug_/average CellTiterGlo_control_).

### 2.6 Knockdown studies

Cells were plated at a density of 3 × 10^5^ cells per well in a 6‐well plate. After 24 h, *Trans*IT‐TKO transfection reagent was added (Mirus Cat no. MIR 2150), followed by CDK13 siRNAs “5,” “10” (product identification numbering system used by Qiagen), and a non‐silencing control (Qiagen Cat no. 1027310), following the Mirus siRNA Transfection Protocol. siRNA “5” had the following target sequence: TCGATTGTATAGCTCAGAAGA (Qiagen Cat. No. 1027417). siRNA “10” had the following target sequence: AACGACGTAGTTTCATTGGAA (Qiagen Cat. No. 1027417). Cells were incubated for 72 h and were then harvested.

To confirm CDK13 knockdown, RNA was extracted from 9 × 10^5^ cells for each siRNA using the RNEasy® Mini Kit protocol (Qiagen) and quantified using the Nanodrop 1000 Spectrophotometer (Thermo Scientific). RNA was converted to cDNA by reverse transcription, following the protocol from the ABI High‐Capacity cDNA Reverse Transcription Kit (Thermo Fisher Scientific). All qPCRs were run on an ABI ViiA 7 Real‐Time PCR System with QuantStudio™ software using 2× qPCRBIO SyGreen Blue Mix, Lo‐ROX (PCR Biosystems). CDK12 and CDK13 primers were designed using the Integrated DNA Technologies PrimerQuest™ Tool. GAPDH primers were designed through the Thermo Fisher Scientific Custom Standard DNA Oligos feature. Primer sequences were as follows: CDK12 Forward: 5′‐CAG GAG AGG CTC TTT GAT TT‐3′, CDK12 Reverse: 5′‐GCC ACT CTG TTT CCC TTA TAG‐3′, CDK13 Forward: 5′‐CAT GGA GGG TCT GGA TTA TTG‐3′, CDK13 Reverse: 5′‐CCG ACT TTC TTC TGA GCT ATA C‐3′, GAPDH Forward: 5′‐AGCCACATCGCTCAGACAC‐3′, GAPDH Reverse: 5′‐GCCCAATACGACCAAATCC‐3′. Analysis of qPCR data was conducted using GraphPad Prism (La Jolla, CA, USA) (GraphPad Prism, RRID:SCR_002798).

Following treatment with siRNAs, cells were plated at 3 × 10^3^ cells per well in 50 μL media. The CDK12 inhibitor BSJ‐4‐116 was prepared at a 10 mm stock solution in DMSO. Using a nine‐point dose curve with a dilution factor of 3, the cells were either a control group or treated at a starting dose of 50 μm. Dose response curves were conducted with five replicates per dose. Plates were imaged at 0 and 72 h of treatment using the ImageXpress Pico live cell imaging system. After 72 h, cell viability was estimated using the CellTiter‐Glo Luminescent Cell Viability Assay kit (Promega) per manufacturer protocol. Luminescence values were measured using the Varioskan Lux plate reader (Thermo Fisher Scientific). Percent viability was calculated as follows: 100*(average CellTiterGlo_drug_/average CellTiterGlo_control_).

### 2.7 Western blots

CRC PDO 4 cells were plated at 5 × 10^4^ cells per well of a 24‐well plate. THZ531, HQ461, and BSJ‐4‐116 were prepared as described above, and cells were treated with one of the following conditions for 24 h: control, 200 nm, 1 μm, or 5 μm of one of the three compounds. HT29 and HCT15 cells were plated at 5 × 10^4^ cells per well of a 24‐well plate. THZ531 and CT7439 were prepared as described above, and cells were treated with one of the following conditions for 24 h: control, 100 nm, 200 nm, or 1 μm of one of the two compounds. CRC cell line 1 cells were plated at 5 × 10^4^ cells per well. The siRNA‐mediated knockdown of CDK13 was administered as described above for a duration of 48 h. Cells were treated with 2.5 μm HQ461 for 24 h. Cells were harvested, then lysed with RIPA buffer and protease/phosphatase inhibitor for 30 min at 4 °C. Solutions were then centrifuged at 15,000 rpm for three minutes and pellets were discarded. Samples were quantified by following the Pierce™ BCA Protein Assay Kit protocol, with approximately 50 μg loaded per well (Thermo Scientific).

All samples were prepared with 4× Laemmli buffer mixed with 2‐mercaptoethanol and heated at 95 °C for five minutes. Samples were then loaded into a 4–20% gradient gel with the Precision Plus Protein™ Kaleidoscope™ Prestained Protein Standards (Bio‐Rad) and run for approximately one hour at 120 V in tris/glycine/SDS running buffer (Bio‐Rad).

Gels were transferred onto a membrane using the iBlot2 according to manufacturer protocols for the iBlot™ 2 Transfer Stacks (Invitrogen). Membranes were blocked for one hour in a nonfat milk, TBS, Tween‐20 mixture (TBS‐T/Milk) at a concentration of 50 g milk and 1 mL Tween 20 per 1 L of TBS. Membranes were washed three times for five minutes each with TBS‐T and incubated at 4 °C in 1 : 1000 solutions of each primary antibody. The RNA polymerase II total antibody was sourced from Active Motif (catalog no. 39097), the phospho‐RNA polymerase II antibody was sourced from Invitrogen (catalog no. MA5‐32637), the cyclin K antibody was sourced from Cell Signaling Technology (catalog no. 19472), and the GAPDH antibody was sourced from Santa Cruz (catalog no. 47724). Membranes were then washed three times for five minutes each with TBS‐T/milk and incubated for one hour at room temperature in 1:3000 dilutions of each appropriate HRP‐linked secondary antibody (Cell Signaling Technology catalog no. 7074P2 and 7076S). Membranes were imaged with the Licor Odyssey Fc imaging system.

### 2.8 BRCA1 qPCR and synergy with CDK12/13 and PARP inhibitors

RKO cells were plated at 3 × 10^5^ cells per well of a 6‐well plate. After 24 h, triplicate wells were treated with 50 nm THZ531, 50 nm BSJ‐4‐116, or control for 24 h. Following treatment, cells were harvested, and RNA extraction, quantification, reverse transcription, and qPCR were performed as described above. BRCA1 exon 1 and BRCA1 exons 20–23 primers were designed using the Integrated DNA Technologies PrimerQuest™ Tool. GAPDH primers were designed through the Thermo Fisher Scientific Custom Standard DNA Oligos feature. Primer sequences were as follows: BRCA1 exon 1 Forward: 5′‐CCT CTG ACT GTG TCT TGA TTT C‐3′, BRCA1 exon 1 Reverse: 5′‐TTA GCT TCC TCG GAA GGA C‐3′, BRCA1 exons 20–23 Forward: 5′‐GGT GAA GGA GCT TTC ATC AT‐3′, BRCA1 exons 20–23 Reverse: 5′‐TGT CCA ACA CCC ACT CT‐3′. GAPDH primer sequences are the same as those used for the CDK13 knockdown in section 2.7. Fold change analysis was accomplished by calculating ΔΔCt values as normalized to the housekeeping (GAPDH) control.

For CDK12/13 and PARP inhibition synergy experiments, cells were plated at 3 × 10^3^ cells per well of a 96‐well plate. THZ531 and CT7439 were prepared at a 10 mm stock solution in DMSO, and olaparib at a 50 mM stock solution in DMSO. Cells were treated with vehicle or one of the following: THZ531 in a nine‐point dose curve with a starting dose of 50 μm and a dilution factor of three; CT7439 in a nine‐point dose curve with a starting dose of 1 μm. Each treatment condition was co‐treated with a five‐point dose curve of olaparib at a starting dose of 50 μm with a dilution factor of five, and each experiment was replicated 1–3 times, depending on cell line. Each dose pair was administered in triplicate, and cells were imaged at 0 and 72 h of treatment, with cell viability estimated after 72 h. Imaging and cell viability estimation were conducted using the method described above. ZIP synergy scores and percent cell inhibition were calculated using SynergyFinder+. ZIP scores were the primary metric used to define synergy for this study, with meaningful synergy being indicated by a ZIP score > 10.

### 2.9 Immunofluorescence assay

RKO cells were plated at a density of 5 × 10^4^ cells per well of a 24‐well plate. CT7439 and olaparib were prepared as described above, and cells were treated with one of the following conditions for 24 h: control, 50 nm CT7439, 50 μm olaparib, or a combination of CT7439 and olaparib. Cells were fixed with 4% paraformaldehyde, permeabilized with 0.2% Triton X‐100 in PBS, blocked with 5% bovine serum albumin (BSA) in PBS, and incubated at 4 °C overnight with a γH2AX or Rad51 primary antibody (Millipore Sigma catalog no. 05–636, SelleckChem catalog no. F1110) at a 1:1000 dilution in 5% BSA. Cells were then incubated for one hour at room temperature with a secondary antibody/Alexa Fluor® 488 conjugate (Cell Signaling Technology catalog no. 4408S and 4412S) and Hoechst nucleic acid stain (Thermo Scientific™ catalog no. 62249) at 1 : 2000 in 5% BSA. Cells were imaged using the Olympus IX73 microscope. Signal intensity was quantified using the green channel analysis parameters on the Sartorius Incucyte®. The set threshold was 2000 GCU, the segmentation parameter was Surface Fit, and all intensity signal was normalized to phase confluence.

### 2.10 Statistical analyses

**Fig. 1:**
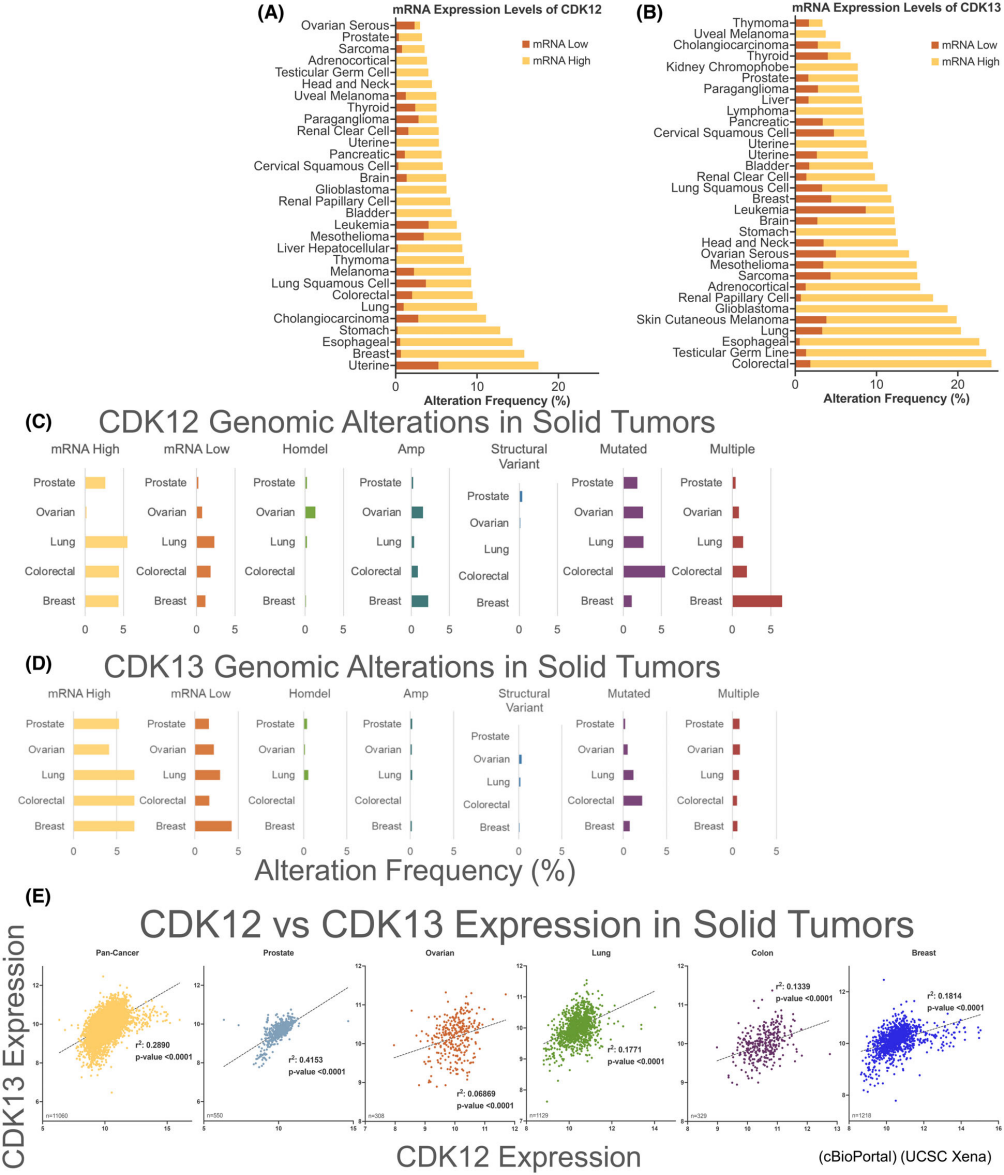
CDK12 and CDK13 expression in solid tumors. (A) mRNA expression levels of CDK12 according to tumor type across a pan‐cancer panel from TCGA. Expression levels are organized by increasing mRNA level. (B) mRNA expression levels of CDK13 according to tumor type across a pan‐cancer panel from TCGA. Expression levels are organized by mRNA level. (C) Common genomic alterations of CDK12 found in a panel five solid tumor types from TCGA: breast, colorectal, lung, ovarian, and prostate. (D) Common genomic alterations of CDK13 found in a panel five solid tumor types from TCGA: breast, colorectal, lung, ovarian, and prostate. (E) Correlation of CDK12 and CDK13 expression from TCGA database cancer patients across five solid tumor types: breast, colorectal, lung, ovarian, and prostate.

IC_50_ values were measured for each dose response curve (Figs 3, 4, and 7), and percent viability was estimated as described above. GraphPad Prism (La Jolla, CA, USA) (GraphPad Prism, RRID:SCR_002798) was used to plot percent viability values as a nonlinear curve fit with the log(inhibitor) vs. response (3 parameters) function. Cell counts for EpCAM staining (Fig. 3) were obtained using the apoptosis analysis feature on the ImageXpress Pico live cell imaging system. Graphs were made using the simple linear regression feature in GraphPad Prism (La Jolla, CA, USA) (GraphPad Prism, RRID:SCR_002798). A one‐way ANOVA analysis was performed in GraphPad Prism to determine significance across cancer types (Fig. 1). *P*‐values < 0.05 were considered statistically reliable. IC_50_ values (Fig. 5) or fold change (Figs 5, 6, and 8) were calculated for each technical replicate, then plotted on a bar graph using GraphPad Prism.

### 2.11 Study approval

Human studies in this manuscript are approved under Duke University IRB protocol Pro000089222. Informed consent was received from human patients before enrollment and participation. Animal studies in this manuscript are approved under Duke University IACUC protocol A023‐24‐01.

## 3 Results

### 3.1 CDK12 and CDK13 are commonly upregulated in solid tumors

CDK12 loss of function can be observed in up to 6% of cancers and can result in a vulnerability to other therapeutics, such as CDK4/6 or PARP inhibition [21]. In addition to this genomic loss, we also identified within TCGA data patient subsets with upregulation or copy gain of CDK12 or CDK13. This percentage ranged from approximately 0.6–22% of samples, depending on cancer type. CDK12 was most commonly upregulated in gastric (12.6%), esophageal (13.8%), and breast (15.2%) cancers while CDK13 was most commonly upregulated in esophageal (22.1%), testicular (22.1%), and colorectal (22.3%) cancers (Fig. 1A,B). Thresholds and cutoffs are described in the Methods section. Comparison of different types of alterations, including mRNA expression, deletions, amplifications, and mutations across the five most common solid tumor types (breast, colon, lung, ovarian, and prostate), revealed mRNA upregulation as the most frequent type of CDK12 and CDK13 alteration (Fig. 1C,D), and CDK12 and CDK13 mRNA expression was positively correlated across cancers (*r* ^2^ = 0.2890 for pan‐cancer dataset, *P* < 0.0001), indicating the potential for similar regulation in their expression (Fig. 1E).

### 3.2 Covalent inhibitors are effective for CDK12 inhibition

**Fig. 2:**
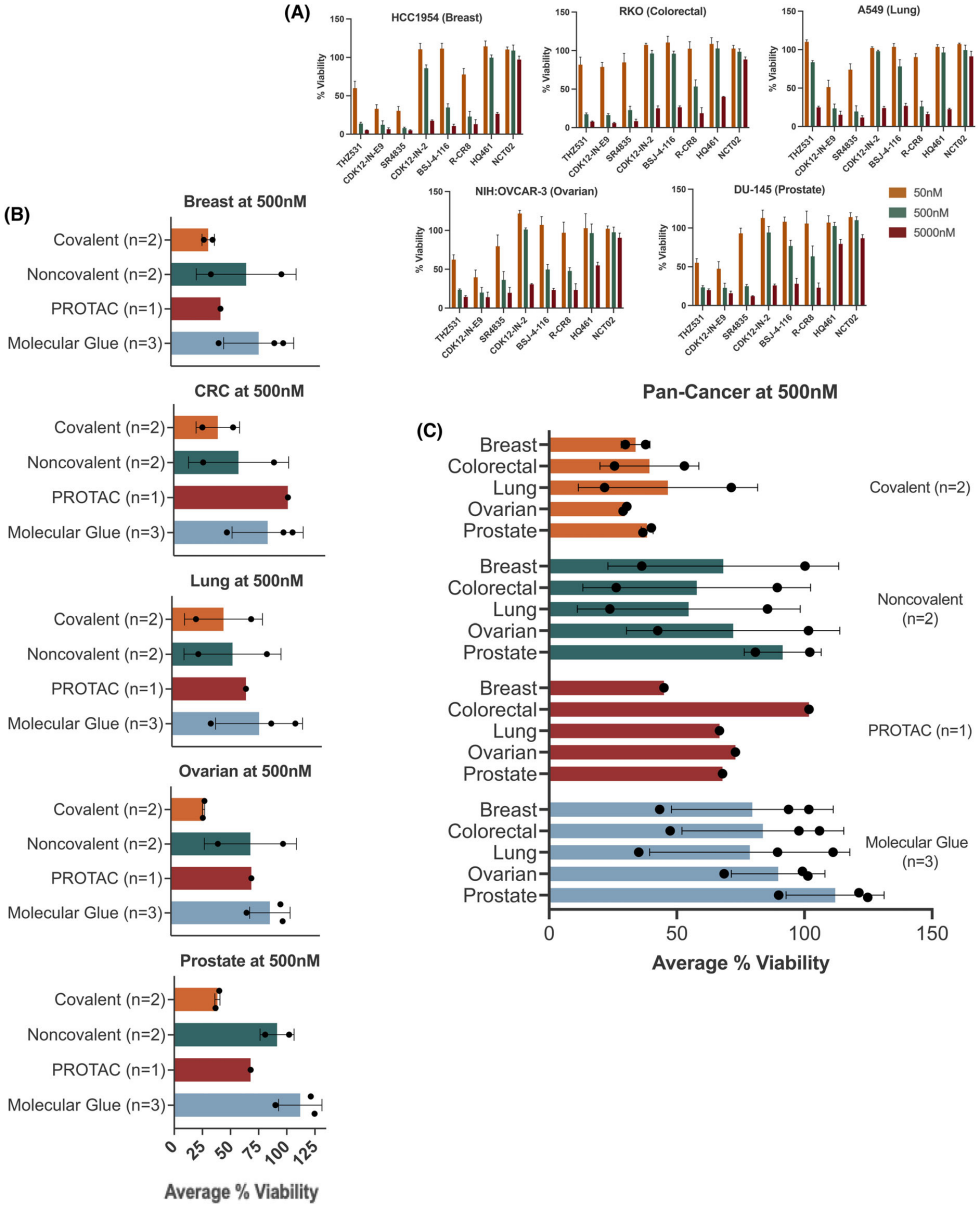
Three‐point dose screening for CDK12/13 inhibition in cell lines. (A) A representative panel of cell lines spanning five solid tumor types was screened with eight CDK12/13 inhibitors (THZ531, CDK12‐IN‐E9, SR4835, CDK12‐IN‐2, BSJ‐4‐116, R‐CR8, HQ461, and NCT02) at 5000 nm, 500 nm, and 50 nm: HCC1954, RKO, A549, NIH:OVCAR‐3, and DU‐145. Error bars indicate SEM. (B) Cell viability of all cell lines screened in Fig. 2A at the middle and most representative dose of 500 nm. Bar graphs are organized by mechanism of drug. Error bars indicate SEM. (C) Cell viability of all cell lines screened in Fig. 2A at the middle and most representative dose of 500 nM. Bar graphs are organized by tumor type. Error bars indicate SEM.

The analysis of public genomics data suggests that CDK12 and CDK13 upregulation is common across multiple cancer types and may represent a potential avenue for therapeutic intervention. To understand the therapeutic potential of this upregulation, we screened a panel of eight CDK12 or CDK12/13 inhibitors with varying mechanisms of inhibition, including covalent (*n* = 2), noncovalent (*n* = 2), PROTAC (*n* = 1), and molecular glue (*n* = 3) across 22 cancer cell lines (breast (*n* = 9), colorectal (*n* = 3), lung (*n* = 3), ovarian (*n* = 2), and prostate (*n* = 5)) (Fig. 2A, Table S1A). Among the various classes of CDK12 or CDK12/13 targeting agents, covalent inhibitors (THZ531 and CDK12‐IN‐E9) were the most effective for this assay, with average percent growth inhibition ranging from 28.6% to 78.3% across all cancer types. Though not explored in this study, THZ531 has been shown to have some effect on other CDKs, such as CDK7 and CDK9. Thus, there is the potential for off‐target effects with this inhibitor [22]. By contrast, the molecular glues (R‐CR8, HQ461, and NCT02) were the least effective for this assay, with average percent growth inhibition ranging from −24.8% to 64.9% across all cancer types. The noncovalent inhibitors (SR4835 and CDK12‐IN‐2) were also found to be inhibitory across all cancer types, though not as efficacious as the covalent inhibitors, while the single PROTAC agent (BSJ‐4‐116) was most effective in breast cancer models (55.0% inhibition) (Fig. 2B,C, Fig. S1A,B).

### 3.3 Validation of CDK12 inhibition in patient‐derived organoids

**Fig. 3:**
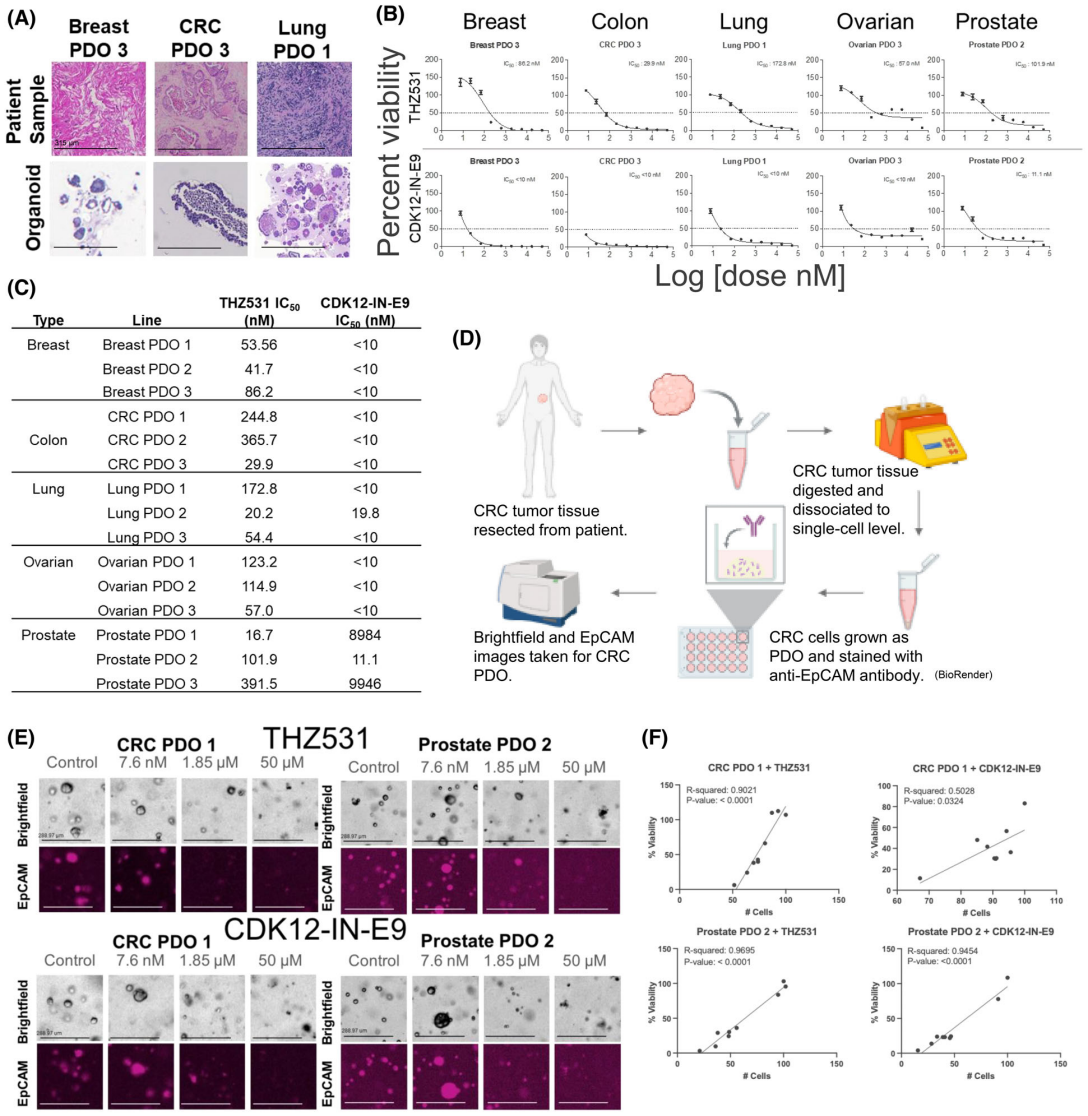
Validation of covalent CDK12/13 inhibition in PDO panel across five solid tumor types. (A) Hematoxylin and eosin (H&E) imaging of patient tumor sample (top panels) and *in vitro* PDO (bottom panels) to confirm PDO resemblance to original patient sample. Scale bar indicates 315 μm. (B) IC_50_ dose–response curves for PDO panel across five solid tumor types: breast, colorectal, lung, ovarian, and prostate. Each sample was treated with THZ531 and CDK12‐IN‐E9 on a nine‐point dose curve from 50 μm to 7.6 nm. Error bars indicate SEM. (C) IC_50_ values from lines treated in Fig. 3A ranged from 391.5 nm to 16.7 nm for THZ531 and 9946 nm to < 10 nm for CDK12‐IN‐E9. (D) Schematic demonstrating the generation of PDO from patient tumors, as well as the EpCAM staining and imaging process. (E) CRC PDO 1 and Prostate PDO 2 were imaged with brightfield and EpCAM imaging at 50 μm, 1.85 μm, 7.6 nm, and control after 72 h of treatment with either THZ531 or CDK12‐IN‐E9. Scale bar indicates 288.97 μm. (F) Cell viability as measured by CTG and EpCAM were correlated for CRC PDO 1 and Prostate PDO 2. *P*‐values were < 0.0001 for treatment with THZ531 and < 0.05 for treatment with CDK12‐IN‐E9.

As the covalent inhibitors were found to be most efficacious in our cell line screen, we sought to further determine the efficacy of this class of drugs using the covalent binders, THZ531 and CDK12‐IN‐E9, in PDO. Samples were first histologically confirmed to contain cancer cells by hematoxylin and eosin (H&E) staining in matching patient tissue and PDO (Fig. 3A). Using CellTiter Glo® assays, IC_50_ curves were generated for 15 PDO lines from breast (*n* = 3), colon (*n* = 3), lung (*n* = 3), ovarian (*n* = 3) and prostate (*n* = 3) cancers (Table 1). All five tumor types were found to be sensitive to THZ531 (IC_50_ = 16.7 nm–391.5 nm), and all tumor types except prostate cancer were found to be sensitive to CDK‐12‐IN‐E9 (IC_50_ = < 10 nm–20 nm) (Fig. 3B,C, Fig. 2A,B).

**Table 1:** Patient‐derived organoid panel. (A) Description of each PDO line, including manuscript‐specific identification, original identification from Duke University BRPC, and solid tumor type.

PDO ID	Duke BRPC ID	Tumor type
CRC PDO 1	CRC119	Colon
CRC PDO 2	BRPC 21‐AEJ	Colon
CRC PDO 3	SD 22–670	CRC liver met
CRC PDO 4	BRPC 21‐AEH (BRPC 21–117)	Colon
CRC PDO 5	SD 22–718	CRC liver met
CRC PDO 6	CRC409	Colon
CRC PDO 7	BRPC 20–423	Colon
CRC PDO 8	CRC240	Colon
CRC PDO 9	SD 23–100	Colon
CRC PDO 10	CRC404	Colon
CRC PDO 11	SD 21–208	Colon
CRC PDO 12	BRPC 21–342 (SD 21–249)	CRC liver met
CRC PDO 13	SD 22–821	CRC liver met
CRC PDO 14	SD 23–138	Colon ovary met
CRC PDO 15	CRC418	Colon
CRC PDO 16	128G	Rectal
CRC PDO 17	SD 22–460	Colon
CRC PDO 18	BRPC 19–192	Colon
CRC PDO 19	BRPC 19–106	Colon
CRC PDO 20	BRPC 20–470	Colon liver met
CRC PDO 21	PM116	CRC liver met
Breast PDO 1	SD 22–667	Breast
Breast PDO 2	SD 23–101	Breast
Breast PDO 3	SD 23–192	Breast
Lung PDO 1	SD 22–711	Colon lung met
Lung PDO 2	SD 23–209	Lung
Lung PDO 3	SD 23–282 (502668)	NSCLC
Ovarian PDO 1	Anonymous	Ovary, pleural effusion
Ovarian PDO 2	SD 23–847	Ovary
Ovarian PDO 3	SD 23–849	Ovary
Prostate PDO 1	SD 23–839	Prostate
Prostate PDO 2	SD 23–924	Prostate
Prostate PDO 3	SD 23–833	Prostate

We next implemented a novel PDO imaging platform that utilizes EpCAM staining to longitudinally track PDO growth inhibition in response to targeted therapies (Fig. 3D). EpCAM is a well‐characterized marker of tumor‐propagating or cancer stem‐like cells [23] and is used as a marker to identify and isolate circulating tumor cells from liquid biopsies [24, 25]. Consistent with our results using CTG, real‐time imaging of PDO growth using EpCAM showed substantial growth inhibition upon treatment with THZ531 or CDK12‐IN‐E9 (Fig. 3E, Fig. 2C). Furthermore, the quantification of EpCAM‐positive cells positively correlated with CTG luminescence values, demonstrating the ability of the EpCAM staining platform to longitudinally follow growth inhibition for cancer cells (Fig. 3F).

### 3.4 Colorectal PDO are sensitive to covalent CDK12 inhibition

**Fig. 4:**
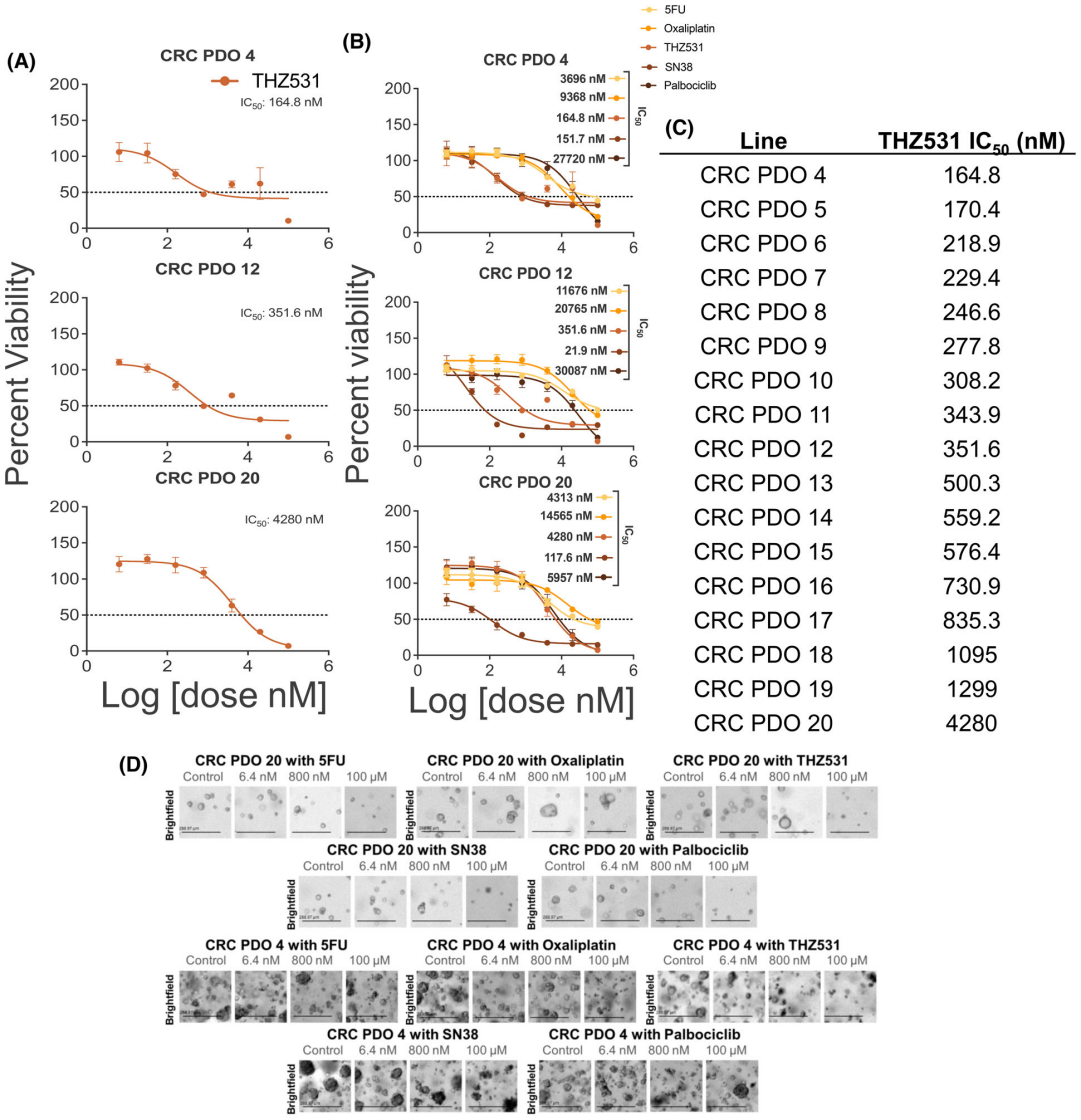
Validation of covalent CDK12/13 inhibition in expanded colorectal cancer PDO panel. (A) IC_50_ dose–response curves for three CRC PDO lines. Each sample was treated with THZ531 on a seven‐point dose curve from 100 μm to 6.4 nm. Error bars indicate SEM. (B) IC_50_ dose–response curves for three CRC PDO lines. Each sample was treated with 5‐Fluorouracil, Oxaliplatin, THZ531, SN38, and Palbociclib on a seven‐point dose curve from 100 μm to 6.4 nm. (C) IC_50_ values from lines treated in Fig. 4A ranged from 4280 nm to 164.8 nm for THZ531. (D) All PDO lines were imaged with brightfield imaging at 100 μm, 800 nm, 6.4 nm, and control after 72 h of treatment with either 5‐Fluorouracil, Oxaliplatin, THZ531, SN38, or Palbociclib. Scale bar indicates 300 μm.

Although CDK12 inhibition is known to be effective in breast and ovarian cancer, its utility in CRC remains unknown. Therefore, we next examined the potential of CDK12 as a therapeutic target in CRC. We further expanded our study's treatment cohort to a panel of 15 CRC PDO (Table 1). Drug sensitivity assays were then performed comparing THZ531 to standard of care chemotherapy used in the treatment of colorectal cancer (oxaliplatin, irinotecan/SN38, and 5‐FU). Palbociclib (CDK4/6 inhibitor) was used as a negative control, given that it has not proven efficacious as a single‐agent treatment in colorectal cancer [26]. In our panel of CRC PDO, IC_50_ values for THZ531 ranged from 164.8 nm to ~4 μm. We also found that CRC PDO were more resistant to oxaliplatin, 5‐FU, and palbociclib, and were more sensitive to irinotecan/SN38 (Fig. 4A–D, Fig. 3A). Together, these results suggest that, in the preclinical setting, CDK12 inhibitors exhibit similar cell growth inhibition as standard of care agents and, in many cases, outperform standard of care chemotherapies in the context of our study.

### 3.5 CDK13 compensates for CDK12 inhibition

**Fig. 5:**
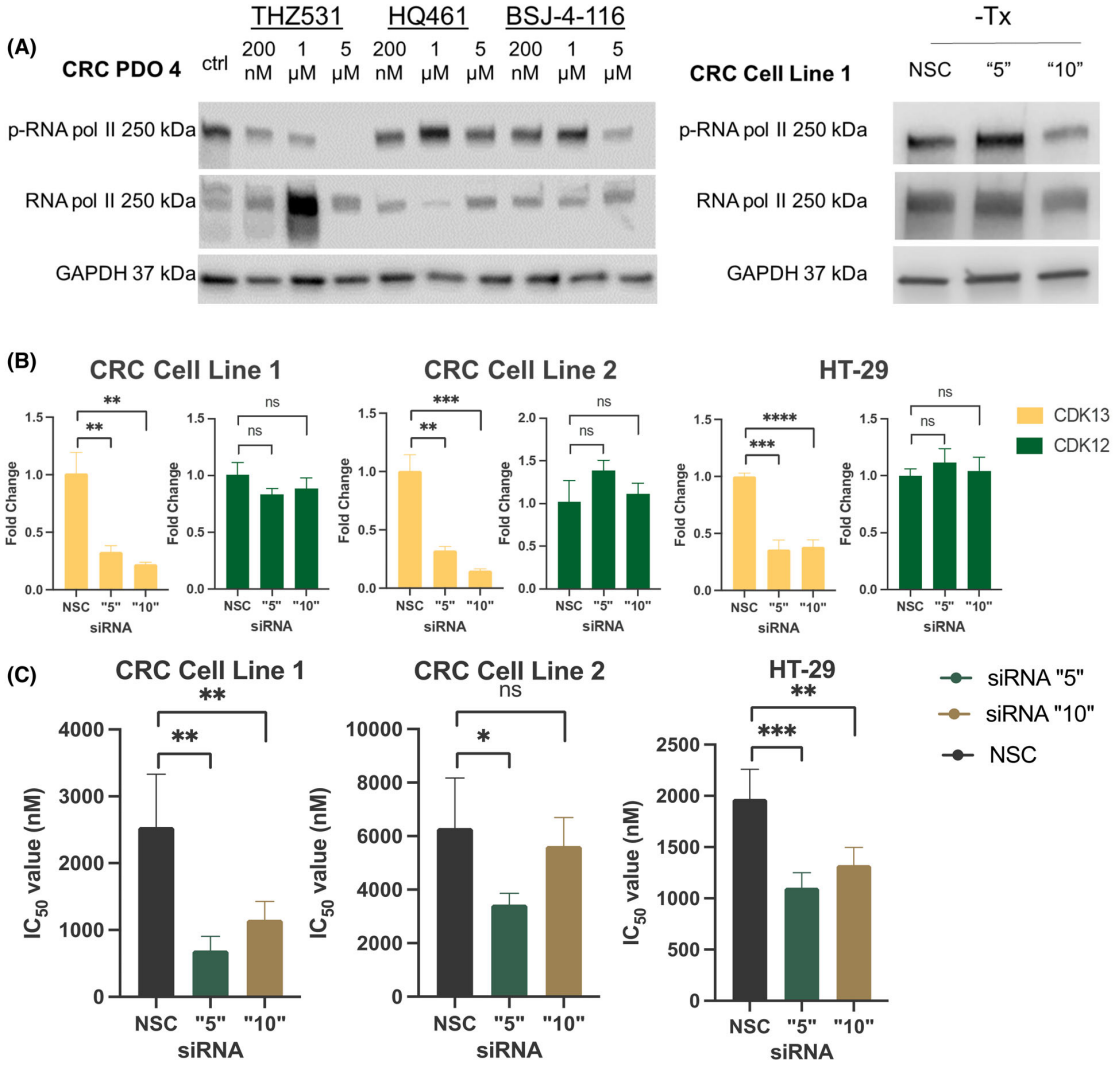
Knockdown of CDK13 sensitizes colorectal cancer cell lines to CDK12 inhibition. (A) Levels of RNA pol II and phospho‐RNA pol II protein in CRC PDO 4 were measured by western blot across the following 24‐h treatment conditions: control, THZ531 (200 nm), THZ531 (1 μm), THZ531 5 μm, HQ461 (200 nm), HQ461 (1 μm), HQ461 (5 μm), BSJ‐4‐116 (200 nm), BSJ‐4‐116 (1 μm), or BSJ‐4‐116 (5 μm). Levels of RNA pol II and phospho‐RNA pol II protein in CRC Cell Line 1 were measured following siRNA‐mediated knockdown of CDK13 for 48 h. (B) siRNAs were used to knock down CDK13 in three colorectal cancer cell lines: CRC cell line 1, CRC cell line 2, and HT‐29. Error bars indicate SEM. Unpaired t‐tests were used to generate *P*‐values. **, ***, and **** represent *P*‐values ≤ 0.01, ≤ 0.001, and ≤ 0.0001, respectively. (C) IC_50_ values of colorectal cancer cell lines treated with CDK12‐specific inhibitor BSJ‐4‐116 after CDK13 knockdown. Cells were treated on a nine‐point dose curve from 50 μm to 7.6 nm. Error bars were determined from the five technical replicates at each dose. Error bars indicate SEM. Unpaired t‐tests were used to generate *P*‐values. *, **, and *** represent *P*‐values ≤ 0.05, ≤ 0.01, and ≤ 0.001, respectively.

CDK13 has been shown to perform an overlapping role to CDK12 [9]. Based on this, we hypothesized that CDK13 expression may compensate for CDK12 inhibition. In support of this hypothesis, protein levels of phospho‐RNA pol II decreased in a dose‐dependent manner following dual CDK12/13 treatment with THZ531, but not with CDK12‐specific treatment. To further test if CDK13 may compensate for CDK12, we used two independent siRNAs to knock down CDK13. CDK13‐specific knockdown alone had no impact on phosphorylated or total RNA pol II (Fig. 5A, right). Using qPCR, we confirmed knockdown of CDK13, as well as the specificity of the siRNAs to only CDK13 and not CDK12 (Fig. 5B). Consistent with an overlapping role for CDK12 and CDK13, siRNA‐mediated knockdown of CDK13 increased the efficacy of the CDK12‐specific inhibitor, BSJ‐4‐116 (Fig. 5C). These results suggest that CDK13 may compensate for the loss of CDK12 function during CDK12‐specific inhibition, with validation in various models and exploration of potential off‐target effects.

### 3.6 Sensitivity to PARP inhibition is increased in combination with CDK12/13 inhibition

**Fig. 6:**
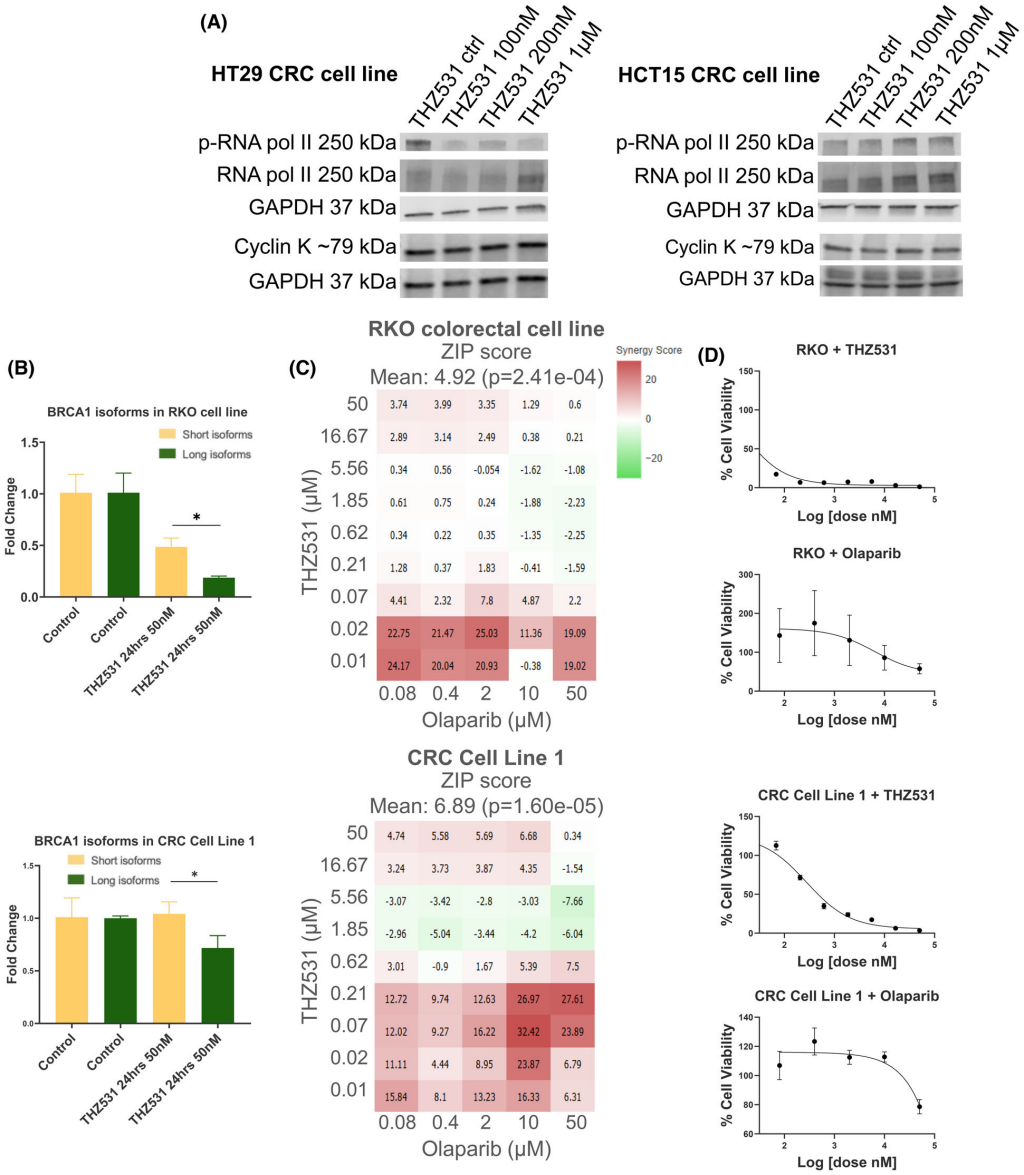
CDK12/13 inhibition synergizes with PARP inhibition in colorectal cancer cell lines. (A) Levels of RNA pol II, phospho‐RNA pol II, and cyclin K proteins in CRC cell lines were measured by western blotting across the following 24‐h treatment conditions: control, THZ531 (100 nm), THZ531 (200 nm), THZ531 (1 μm). (B) RKO and CRC Cell Line 1 colorectal cancer cells were treated with THZ531 at 50 nm for 24 h. qPCR results measuring BRCA1 show a significant decrease in the presence of long isoforms of BRCA1 post‐treatment. Error bars indicate SEM. Unpaired t‐tests were used to generate *P*‐values. * represents *P* ≤ 0.05. (C, D) RKO and CRC Cell Line 1 colorectal cancer cells were treated with THZ531 (nine‐point dose curve from 50 μm to 7.6 nm) and olaparib (five‐point dose curve from 50 μm to 80 nm) for 120 h. Using ZIP scores, synergy is demonstrated at higher doses of both THZ531 and olaparib. Error bars indicate SEM.

Prior studies have demonstrated that CDK12 loss renders cells susceptible to PARP inhibitors by preventing transcription elongation of long transcripts, including BRCA1 and other DNA damage pathway genes [27]. To test if this strategy may be useful in CRC, we first conducted western blots using lysates from HT29 and HCT15 CRC cell lines to assess the protein levels of (p‐)RNA pol II and cyclin K (Fig. 6A). We found that, with THZ531 treatment, p‐RNA pol II levels decrease, but cyclin K remains the same. We treated RKO CRC cells and CRC cell line 1 with the CDK12/13 inhibitor, THZ531, as well as with the CDK12‐specific PROTAC, BSJ‐4‐116. Using qPCR, we found that treatment with THZ531, but not BSJ‐4‐116, led to a significant reduction in long isoforms of BRCA1 (Fig. 6B, Fig. 4A). Consistent with this downregulation of BRCA1, treatment with THZ531 and the PARP inhibitor olaparib led to synergistic cell growth inhibition at low doses (Fig. 6C,D).

### 3.7 Novel CDK12/13 inhibitor CT7439 shows efficacy in CRC PDO

**Fig. 7:**
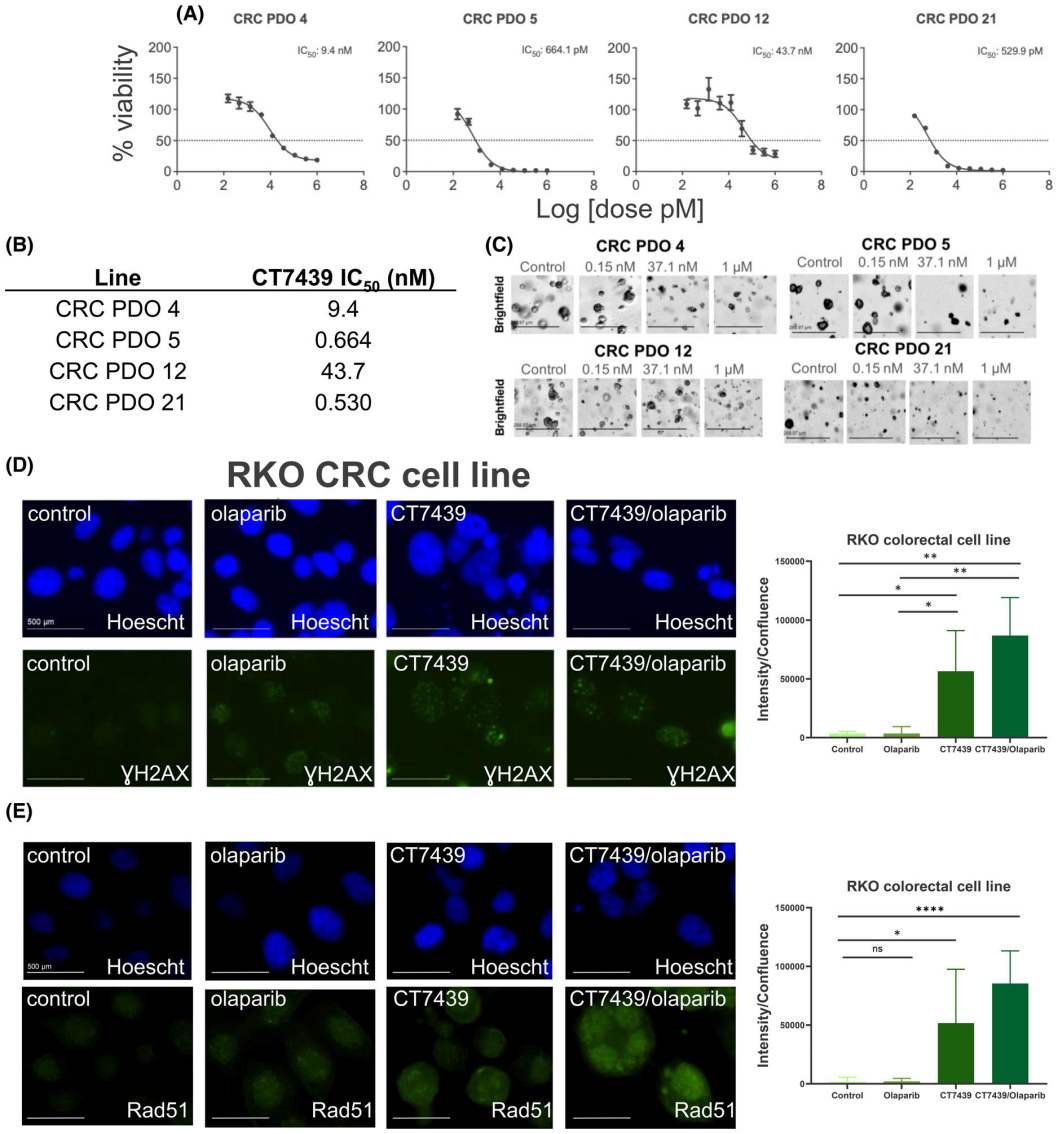
Evaluation of novel CDK12/13 inhibitor, CT7439, for the treatment of CRC. (A) IC_50_ dose–response curves for four CRC PDO lines. Each sample was treated with CT7439 on a nine‐point dose curve from 1 μm to 0.15 nm. Error bars indicate SEM. (B) IC_50_ values from lines treated in Fig. 7A ranged from 43.7 nm to 529.9pm. (C) All PDO lines were imaged with brightfield imaging at 1 μm, 37.1 nm, 0.15 nm, and control after 72 h of treatment with CT7439. (D) RKO, a colorectal cancer cell line, was dosed with one of the following conditions for 24 h: control, 50 nm CT7439, 10 μm olaparib, or 50 nm CT7439 and 10 μm olaparib. ƔH2AX foci were quantified for each image and were normalized to number of cells with a foci/cell number ratio: 0.6 (control), 15.6 (olaparib), 11 (CT7439), 17.5 (olaparib/CT7439). Error bars indicate SEM. Cells were then stained for Hoechst and ƔH2AX and were imaged with immunofluorescent microscopy to assess levels of DNA damage. Levels of DNA damage measured by ƔH2AX were quantified. Scale bars indicated 500 μm. A one‐way ANOVA was used to generate *P*‐values. *, **, and **** represent *P*‐values ≤ 0.05, ≤ 0.01, and ≤ 0.0001, respectively. (E) Colorectal cancer cell line RKO was dosed with one of the following conditions for 24 h: control, 50 nm CT7439, 10 μm olaparib, or 50 nm CT7439 and 10 μm olaparib. Cells were then stained for Hoechst and Rad51 and were imaged by immunofluorescence to assess levels of homologous repair deficiency. Levels of homologous repair deficiency measured by Rad51 were quantified. Error bars indicate SEM. Scale bar indicates 500 μm. A one‐way ANOVA was used to generate *P*‐values. *, **, and **** represent *P*‐values ≤ 0.05, ≤ 0.01, and ≤ 0.0001, respectively.

The promising results from multiple CDK12 and CDK12/13 inhibitors across multiple cancer types prompted further evaluation of CT7439, a novel CDK12/13 glue‐degrader, in a panel of CRC PDO (Fig. 7A). CT7439 functions by inhibiting CDK12 and CDK13, as well as degrading cyclin K, allowing this inhibitor to specifically target both CDK12 and CDK13 as well as their obligate cofactor. CT7439 is currently in Phase 1/2 clinical trials in a range of solid tumors [28]. Across a panel of four CRC PDOs, IC_50_ values ranged from 0.53 nm to 43.7 nm, showing potent cell growth inhibition of CRC (Fig. 7B,C). In continuing our exploration into CDK12/13 and PARP inhibition synergy, we assessed levels of DNA damage induced from treatment with CT7439 and olaparib (Fig. 7D, Fig. 5A). Quantification of ƔH2AX intensity normalized to number of cells per well showed significantly higher levels of DNA damage upon treatment with CT7439 or combination of CT7439 and olaparib. We also assessed levels of homologous repair deficiency induced by CT7439 and olaparib (Fig. 7E). Quantification of Rad51 intensity normalized to number of cells per well showed significantly higher levels of homologous repair deficiency with CT7439 or the combination of CT7439 and olaparib.

**Fig. 8:**
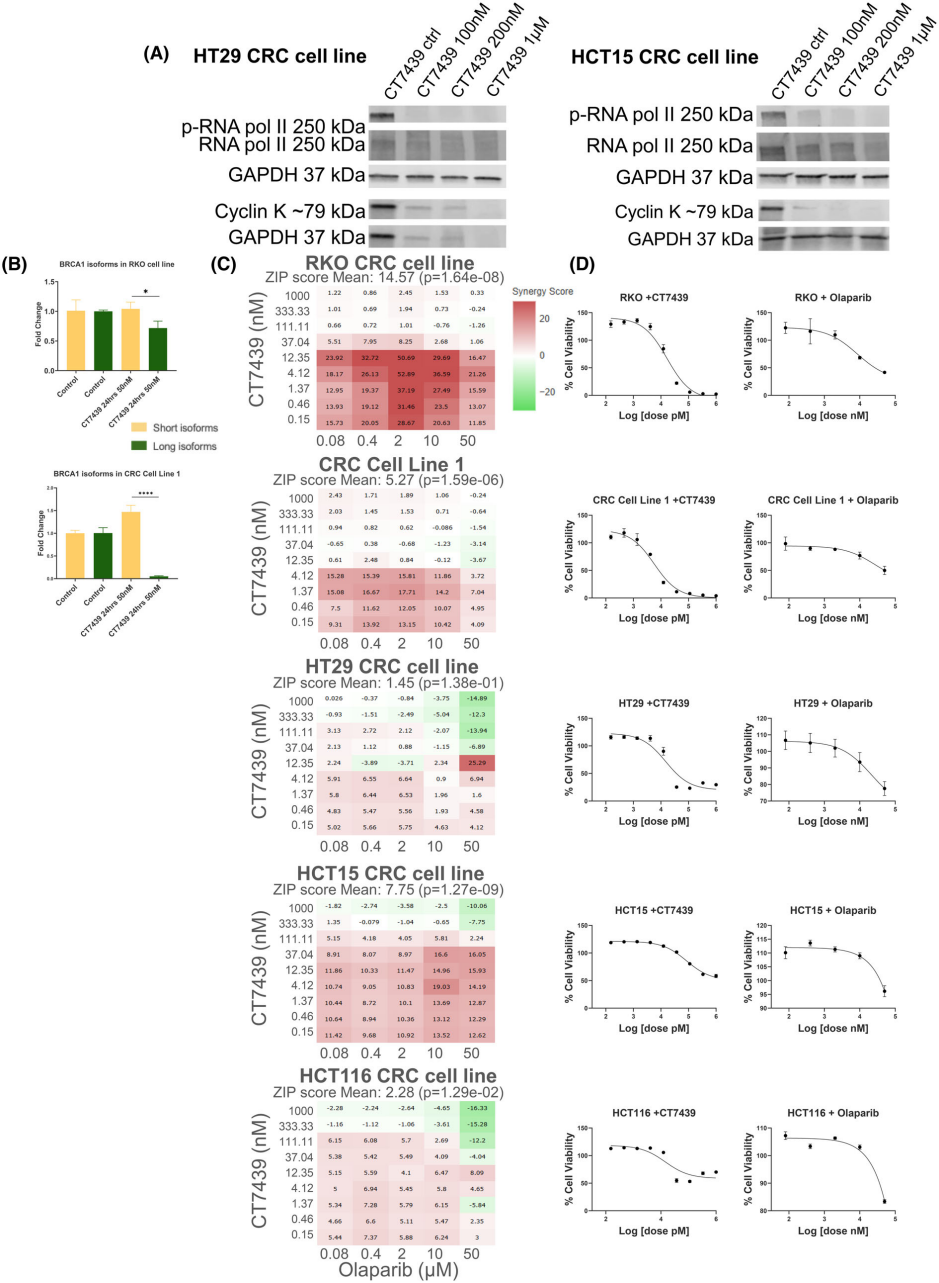
CDK12/13 inhibition with CT7439 synergizes with PARP inhibition in colorectal cancer cell lines. (A) Levels of RNA pol II, phospho‐RNA pol II, and cyclin K proteins in CRC cell lines were measured by western blot across the following 24‐h treatment conditions: control, CT7439 (100 nm), CT7439 (200 nM), CT7439 (1 μm). (B) RKO and CRC Cell Line 1 colorectal cancer cells were treated with CT7439 at 50 nm for 24 h. qPCR results measuring BRCA1 show a significant decrease in the presence of long isoforms of BRCA1 post‐treatment. Error bars indicate SEM. Unpaired t‐tests were used to generate *P*‐values. * and **** represent *P* ≤ 0.05 and *P* ≤ 0.0001, respectively. (C, D) Colorectal cancer cells were treated with CT7439 (nine‐point dose curve from 1 μm to 0.15 nm) and olaparib (five‐point dose curve from 50 μm to 80 nm) for 120 h. Using ZIP scores, synergy is demonstrated at higher doses of both CT7439 and olaparib. Error bars indicate SEM. The proposed mechanism of action for the CDK12/13 inhibitor and cyclin K degrader, CT7439. CDK12/13 inhibition interrupts transcription elongation, leading to increased DNA damage that results in cell death. This agent is a potentially novel treatment option for patients with colorectal cancer. Created in BioRender.

To further test the potential of combination therapy with CT7439, we conducted a western blot on HT29 and HCT15 CRC cell lines to assess the protein levels of (p‐)RNA pol II and cyclin K (Fig. 8A). CT7439 treatment led to reduction in p‐RNA pol II levels, but no change in cyclin K. We treated RKO CRC cells and CRC cell line 1 with the CDK12/13 inhibitor CT7439, as well as with the CDK12‐specific PROTAC, BSJ‐4‐116. Treatment with CT7439, but not BSJ‐4‐116, led to a significant reduction in long isoforms of BRCA1, as measured with isoform‐specific RT‐qPCR assays for short and long BRCA1 isoforms (Fig. 8B, Fig. 6A). Consistent with this downregulation of BRCA1, treatment with CT7439 and the PARP inhibitor olaparib led to synergistic cell growth inhibition at low doses (Fig. 8C,D). Together, these results demonstrate the promise of the novel CDK12/13 inhibitor CT7439 in CRC.

## 4 Discussion

CDK12 loss of function is an important marker for determining sensitivity of cancer cells to treatments such as PARP inhibition [13]. However, while most of the focus on CDK12 is related to its loss of function, we were surprised to discover common upregulation of CDK12 and its closely related paralog, CDK13. As mentioned previously, CDK12 and CDK13 upregulation in cancers can range from 0.6–22% of samples through our analysis of publicly available data from The Cancer Genome Atlas. CDK12 was most commonly upregulated in gastric (12.6%), esophageal (13.8%), and breast (15.2%) cancers while CDK13 was most commonly upregulated in esophageal (22.1%), testicular (22.1%), and colorectal (22.3%) cancers. The upregulation of these closely‐related genes, as well as their functions in transcriptional elongation and cell proliferation, offers a therapeutic vulnerability for cancerous cells. While CDK inhibitors are promising agents for the treatment of solid tumors, therapies with non‐selective CDK inhibitors often have associated toxicities because of the involvement of CDKs in critical processes, such as cell cycle progression and transcription. Using selective inhibitors in precision‐guided strategies, however, may be a path forward. For example, CDK4/6 inhibitors are approved for hormone receptor positive breast cancer because of generations of testing for highly selective but less toxic inhibitors [29]. CDK2/9 inhibitors are being developed to induce anaphase catastrophe [30], and CDK12 loss is a predictive biomarker for the use of PARP inhibition because of its role in transcriptional elongation of long mRNAs in the DNA damage response pathway [31]. In the case of CDK12, its function in phosphorylating the CTD of RNA pol II makes it a necessary protein for global transcription and cell viability [11]. We found CDK12 upregulation to be a common aberration among solid tumors, observed in ~0.6–22% over a range of sample types from The Cancer Genome Atlas. Across a panel of solid tumor models screened in our study, including both cell lines and PDO, chemical inhibition of CDK12/13 using a suite of small molecule CDK12/13 antagonists led to cell growth inhibition.

Among the inhibitors we tested, we found the dual CDK12/13 inhibitors, THZ531 and CT7439, as some of the most potent agents tested. It is important to note the potential limitations of our screens, one of which is the possibility for off‐target effects of these agents that were not tested for in this study. Additionally, we did not test for potential transcriptional toxicity resulting from dual CDK12/13 inhibition. The observed resistance against CDK12‐specific inhibitors [32] suggests a potential role for CDK13 as a compensatory mechanism for CDK12 inhibition. This was supported by our studies using siRNA‐mediated knockdown of CDK13, which improved sensitivity to CDK12 inhibition. Co‐inhibition of both CDK12 and CDK13 has been shown to decrease cell viability of high grade serous ovarian cancers, suggesting that inhibition of CDK12 and CDK13 may be a more effective treatment strategy [33]. However, while dual inhibition of CDK12/13 has effectively been applied to preclinical models, these inhibitors can often be too toxic for clinical use [27]. Given this toxicity, it may be worthwhile exploring CDK12/13 inhibition in the subsets of patients with CDK12/13 upregulation and with novel agents that have improved selectivity.

As a critical mediator of RNA pol II elongation, CDK12 is essential for proper transcriptional regulation. Loss‐of‐function mutations in CDK12 prevent RNA pol II elongation, leading to premature mRNA polyadenylation [34]. While this can have negative consequences for non‐cancer cells, it also provides a potential therapeutic vulnerability for cancer cells. For example, many genes involved in the DNA damage response have long mRNAs [9], and CDK12 promotes RNA pol II transcription elongation to ensure proper transcription of these DNA damage repair genes [12]. Because of its role in RNA pol II elongation of mRNAs involved in DNA damage repair, CDK12 has gained attention as a potential synthetic lethality therapy with PARP inhibitors [34]. Based on this, CDK12 inhibition in combination with PARP inhibition is emerging as a new potential method of cancer treatment. PARP inhibition alone may not prevent the growth of BRCA wild‐type cells, as these cells do not possess mutations in homologous recombination processes [31]. When CDK12 inhibition is combined with a PARP inhibitor, however, sensitivity has been shown to increase [27]. For example, combined treatment with PP‐C8 (a CDK12‐targeting PROTAC) along with a PARP inhibitor was shown to synergistically inhibit triple negative breast cancer cells [27].

## 5 Conclusions

In summary, we pinpoint the CDK12/13 inhibitor and cyclin K degrader, CT7439, as a promising new therapeutic strategy for CRC and other solid tumors. Currently in Phase 1/2 clinical trials, CT7439 is being assessed for potential toxicity in patients [28]. It is possible that patients with CDK12 upregulation and CDK13 loss of function may benefit most from CDK12 inhibition since normal cells would have CDK13 to compensate for the CDK12 loss. Likewise, the rarer subset of patients with upregulation of both CDK12 and CDK13 may benefit from dual CDK12/13 inhibition. Future clinical trials should consider the underlying genomic features of CDK12 and CDK13 expression in their trial design.

## Conflict of interest

Dr. Ashwani Bahl is CSO at Carrick Therapeutics, Inc. Jeremy Force is an employee with stock options at Daiichi Sankyo, Inc.

## Author contributions

WKW: Data curation, formal analysis, investigation, writing‐original draft, writing‐review and editing. DLD: Data curation, writing‐review and editing. MZ: Data curation, formal analysis, writing‐review and editing. JBM: Data curation, writing‐review and editing. PDO: Data curation. GR: resources, supervision, writing‐review, and editing. JMF: Conceptualization. SJM: Data curation, writing‐review and editing. AKB: Resources, writing‐review and editing. DSH: Conceptualization, supervision, funding acquisition, visualization, methodology, project administration, writing‐review and editing. JAS: Conceptualization, supervision, project administration, writing‐review, and editing.

## Supporting information


**Fig. S1.** Three‐point dose screening for CDK12/13 inhibition in cell lines. (A) Cell viability of all cell lines screened in Fig. 2A at the lowest dose of 50 nm. Bar graphs are organized by mechanism of drug. Error bars indicate SEM. (B) A Cell viability of all cell lines screened in Fig. 2A at the highest dose of 5000 nm. Bar graphs are organized by mechanism of drug. Error bars indicate SEM.
**Table S1**. CDK12/13 inhibitor panel. (A) Description of all CDK12/13 inhibitors in panel, including mechanism of action, molecular weight, inhibition specificity, and cell‐free IC5_0_ as described by MedChemExpress.
**Fig. S2.** Validation of covalent CDK12/13 inhibition in PDO panel across five solid tumor types. (A) IC_50_ dose–response curves for PDO panel across five solid tumor types: breast, colorectal, lung, ovarian, and prostate. Each sample was treated with THZ531 and CDK12‐IN‐E9 on a nine‐point dose curve from 50 μm to 7.6 nm. Error bars indicate SEM. (B) All PDO lines were imaged with brightfield imaging at 50 μm, 1.85 μm, 7.6 nm, and control after 72 h of treatment with either THZ531 or CDK12‐IN‐E9. Scale bar indicates 288.97 μm. (C) Remaining PDO lines were imaged with brightfield and EpCAM imaging at 50 μm, 1.85 μm, 7.6 nm, and control after 72 h of treatment with either THZ531 or CDK12‐IN‐E9. Scale bar indicates 288.97 μm.
**Fig. S3.** Validation of covalent CDK12/13 inhibition in expanded colorectal cancer PDO panel. (A) IC_50_ dose–response curves for all colorectal PDO lines. Each sample was treated with 5‐Fluorouracil, Oxaliplatin, THZ531, SN38, and Palbociclib on a seven‐point dose curve from 100 μm to 6.4 nm. Error bars indicate SEM.
**Fig. S4.** CDK12/13 inhibition synergizes with PARP inhibition in colorectal cancer cell lines. (A) RKO and CRC Cell Line 1 colorectal cancer cells were treated with BSJ‐4‐116 at 50 nm for 24 h and levels of BRCA1 isoforms were measured via qPCR. Error bars indicate SEM. Unpaired t‐tests were used to generate *P*‐values. “ns” represents *P* > 0.05.
**Fig. S5.** Screening of novel CDK12/13 inhibitor CT7439. (A) RKO, a colorectal cancer cell line, was dosed with one of the following conditions for 24 h: control, 50 nm CT7439, 10 μm olaparib, or 50 nm CT7439 and 10 μm olaparib. Merged images of Hoechst and ƔH2AX staining are shown. Scale bar indicates 500 μm.
**Fig. S6.** CDK12/13 inhibition with CT7439 synergizes with PARP inhibition in colorectal cancer cell lines. (A) RKO and CRC Cell Line 1 colorectal cancer cells were treated with BSJ‐4‐116 at 50 nm for 24 h and levels of BRCA1 isoforms were measured via qPCR. Error bars indicate SEM. Unpaired *t*‐tests were used to generate *P*‐values. “ns” represents *P* > 0.05.

## Data Availability

The data sets from the Cancer Genome Atlas via cBioPortal and the University of California, Santa Cruz Xena Browser used and/or analyzed during the current study are available from the corresponding author on reasonable request.
