# Supplementary material for: Inhibition of cyclin‐dependent kinases 12/13 using CT7439 as a treatment for colorectal cancer with CDK12 upregulation
Source: Mol Oncol. 2026 Jun 30:10.1002/1878-0261.70290. Online ahead of print. doi: 10.1002/1878-0261.70290 (PMC13399226; doi:10.1002/1878-0261.70290)
Supplement: Supplementary file 1 — Fig. S1. Three‐point dose screening for CDK12/13 inhibition in cell lines. (A) Cell viability of all cell lines screened in Fig. 2A at the lowest dose of 50 nm. Bar graphs are organized by mechanism of drug. Error bars indicate SEM. (B) A Cell viability of all cell lines screened in Fig. 2A at the highest dose of 5000 nm. Bar graphs are organized by mechanism of drug. Error bars indicate SEM. Table S1. CDK12/13 inhibitor panel. (A) Description of all CDK12/13 inhibitors in panel, including mechanism of action, molecular weight, inhibition specificity, and cell‐free IC50 as described by MedChemExpress. Fig. S2. Validation of covalent CDK12/13 inhibition in PDO panel across five solid tumor types. (A) IC50 dose–response curves for PDO panel across five solid tumor types: breast, colorectal, lung, ovarian, and prostate. Each sample was treated with THZ531 and CDK12‐IN‐E9 on a nine‐point dose curve from 50 μm to 7.6 nm. Error bars indicate SEM. (B) All PDO lines were imaged with brightfield imaging at 50 μm, 1.85 μm, 7.6 nm, and control after 72 h of treatment with either THZ531 or CDK12‐IN‐E9. Scale bar indicates 288.97 μm. (C) Remaining PDO lines were imaged with brightfield and EpCAM imaging at 50 μm, 1.85 μm, 7.6 nm, and control after 72 h of treatment with either THZ531 or CDK12‐IN‐E9. Scale bar indicates 288.97 μm. Fig. S3. Validation of covalent CDK12/13 inhibition in expanded colorectal cancer PDO panel. (A) IC50 dose–response curves for all colorectal PDO lines. Each sample was treated with 5‐Fluorouracil, Oxaliplatin, THZ531, SN38, and Palbociclib on a seven‐point dose curve from 100 μm to 6.4 nm. Error bars indicate SEM. Fig. S4. CDK12/13 inhibition synergizes with PARP inhibition in colorectal cancer cell lines. (A) RKO and CRC Cell Line 1 colorectal cancer cells were treated with BSJ‐4‐116 at 50 nm for 24 h and levels of BRCA1 isoforms were measured via qPCR. Error bars indicate SEM. Unpaired t‐tests were used to generate P‐values. “ns” represents P > 0.05. [file MOL2-9999-0-s001.pdf]

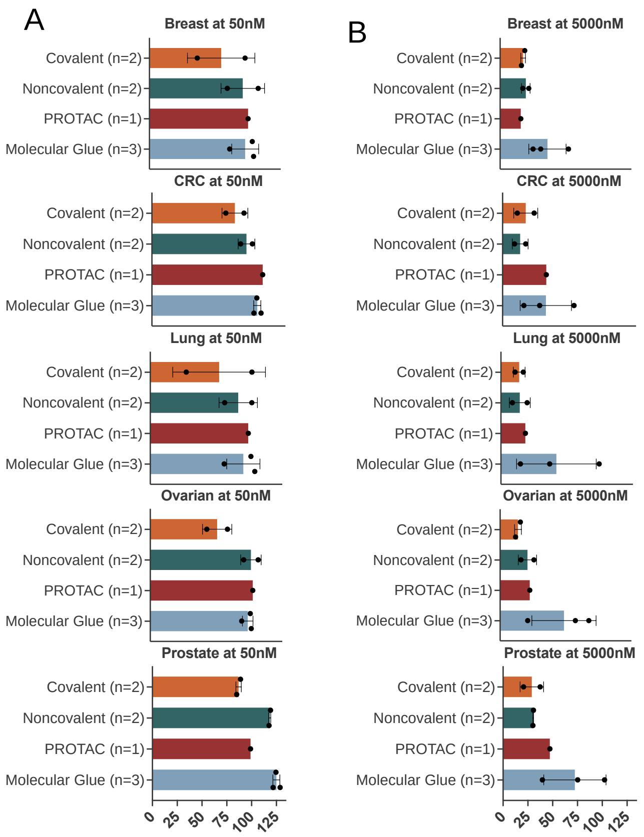

Supplemental Figure 1: Three-point dose screening for CDK12/13 inhibition in cell lines. A) Cell viability of all cell lines screened in Figure 2A at the lowest dose of 50nM. Bar graphs are organized by mechanism of drug. Error bars indicate SEM. B) A) Cell viability of all cell lines screened in Figure 2A at the highest dose of 5000nM. Bar graphs are organized by mechanism of drug. Error bars indicate SEM.

A

Mechanism	Compound	Inhibition of	Cell-free IC_{50}
Covalent	THZ531 (558.07g/mol)	**CDK12**	158nM
		**CDK13**	69nM
	CDK12-IN-E9 (434.53g/mol)	CDK2	932nM
		CDK7	1210nM
		**CDK12**	n/a
Noncovalent	SR4835 (499.36g/mol)	**CDK12**	99nM
		**CDK13**	4.9nM
	CDK12-IN-2 (532.64g/mol)	CDK2	>100μM
		CDK7	>10μM
		CDK9	16μM
		**CDK12**	52nM
		**CDK13**	n/a
PROTAC	BSJ-4-116 (837.38g/mol)	**CDK12**	6nM
Molecular Glue	R-CR8 (431.53g/mol)	CDK1	90nM
		CDK2	72nM
		CDK5	110nM
		CDK7	1100nM
		CDK9	180nM
		**CDK12**	n/a
	HQ461 (345.44g/mol)	**CDK12**	1300nM
	NCT02 (312.39g/mol)	**CDK12**	n/a

(MedChemExpress)

Supplemental Table 1: CDK12/13 inhibitor panel. A) Description of all CDK12/13 inhibitors in panel, including mechanism of action, molecular weight, inhibition specificity, and cell-free IC_{50} as described by MedChemExpress.

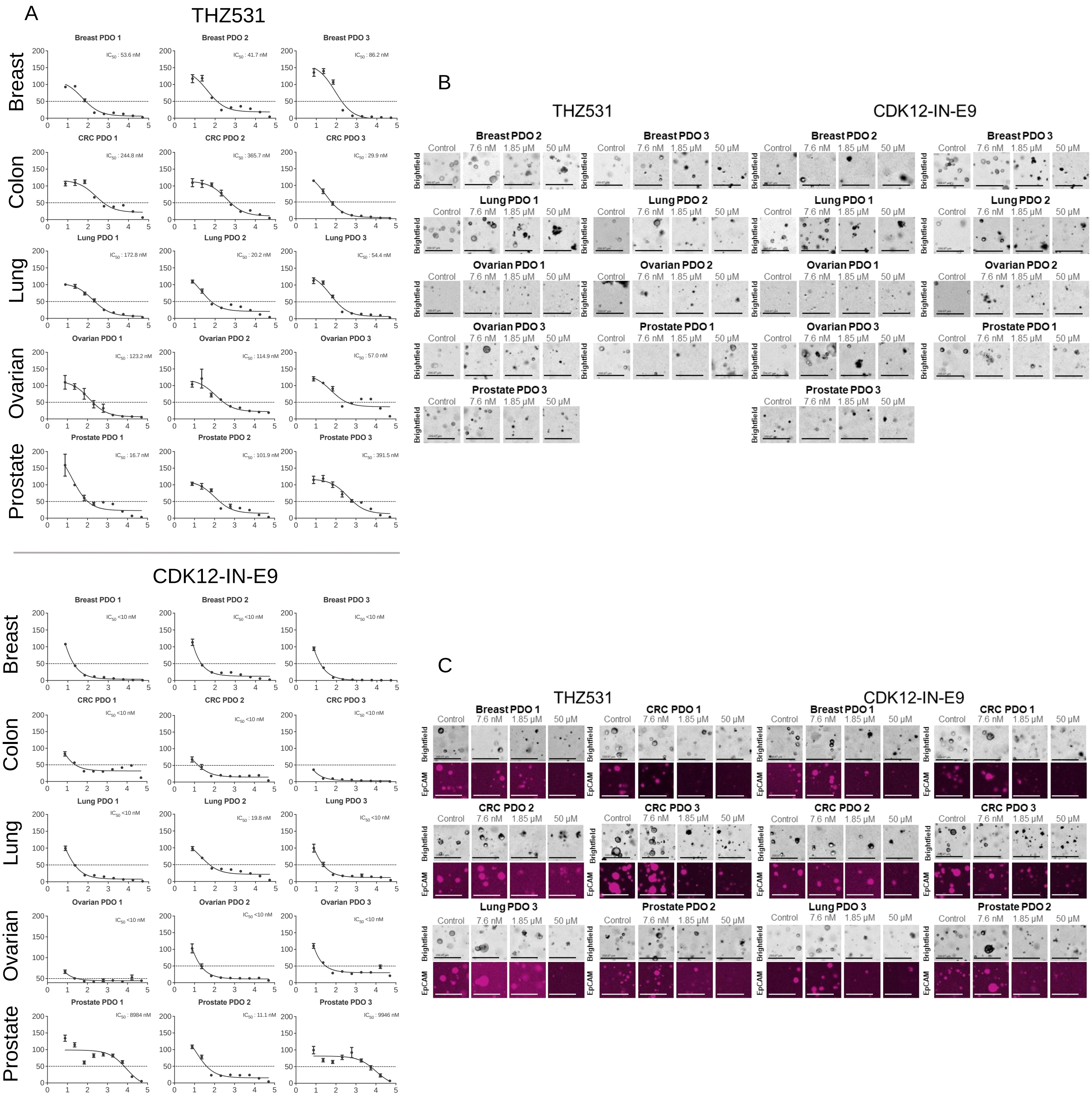

Supplemental Figure 2: Validation of covalent CDK12/13 inhibition in PDO panel across five solid tumor types. A) IC_{50} dose-response curves for PDO panel across five solid tumor types: breast, colorectal, lung, ovarian, and prostate. Each sample was treated with THZ531 and CDK12-IN-E9 on a nine-point dose curve from 50µM to 7.6nM. Error bars indicate SEM. B) All PDO lines were imaged with brightfield imaging at 50µM, 1.85µM, 7.6nM, and control after 72 hours of treatment with either THZ531 or CDK12-IN-E9. Scale bar indicates 288.97µm. C) Remaining PDO lines were imaged with brightfield and EpCAM imaging at 50µM, 1.85µM, 7.6nM, and control after 72 hours of treatment with either THZ531 or CDK12-IN-E9. Scale bar indicates 288.97µm.

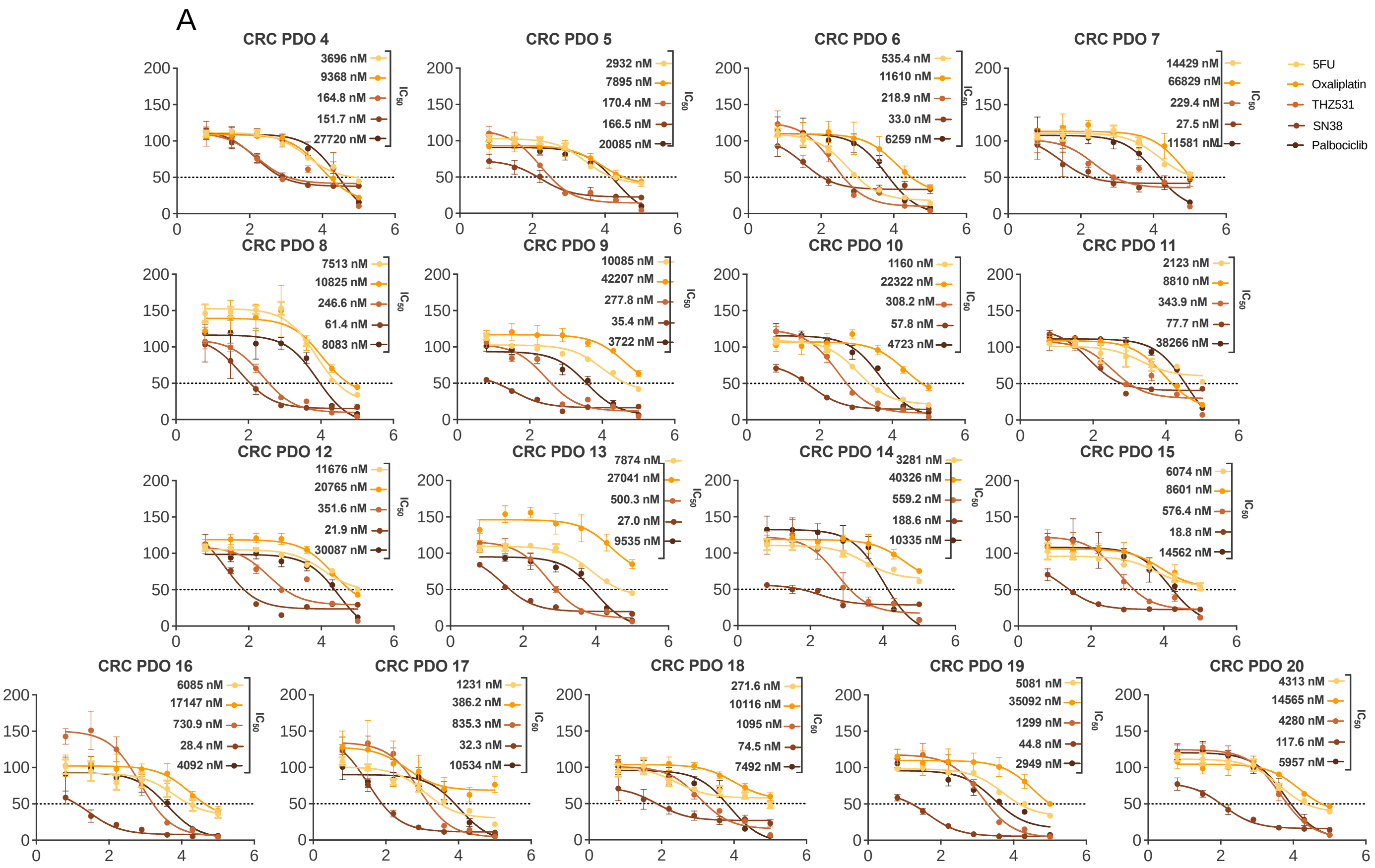

Supplemental Figure 3: Validation of covalent CDK12/13 inhibition in expanded colorectal cancer PDO panel. A) IC_{50} dose-response curves for all colorectal PDO lines. Each sample was treated with 5-Fluorouracil, Oxaliplatin, THZ531, SN38, and Palbociclib on a seven-point dose curve from 100µM to 6.4nM. Error bars indicate SEM.

A

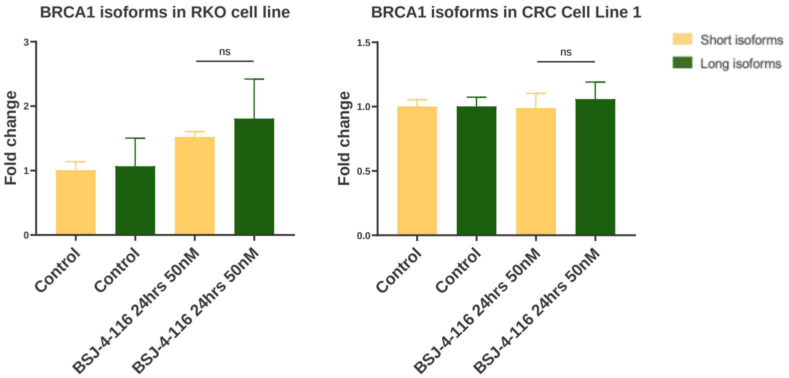

Supplemental Figure 4: CDK12/13 inhibition synergizes with PARP inhibition in colorectal cancer cell lines. A) RKO and CRC Cell Line 1 colorectal cancer cells were treated with BSJ-4-116 at 50nM for 24 hours and levels of BRCA1 isoforms were measured via qPCR. Error bars indicate SEM. Unpaired t-tests were used to generate p-values. “ns” represents p>0.05.

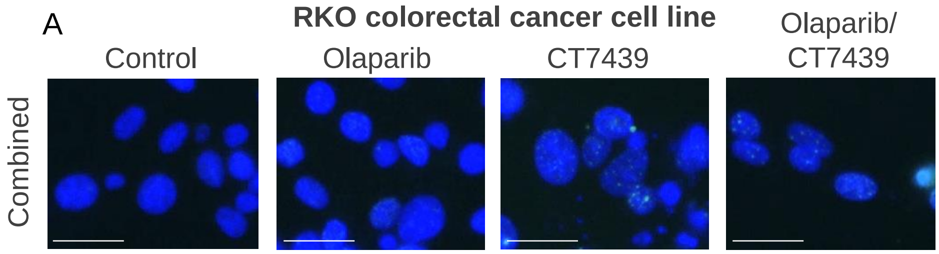

Supplemental Figure 5: Screening of novel CDK12/13 inhibitor CT7439. A) RKO, a colorectal cancer cell line, was dosed with one of the following conditions for 24 hours: control, 50nM CT7439, 10µM olaparib, or 50nM CT7439 and 10µM olaparib. Merged images of Hoechst and ɣH2AX staining are shown. Scale bar indicates 500µm.

A

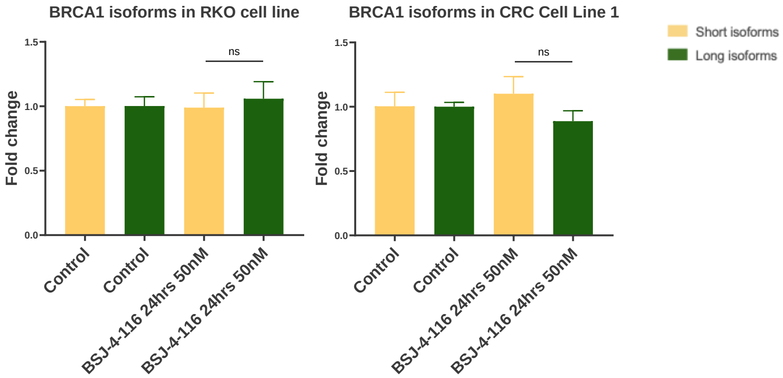

Supplemental Figure 6: CDK12/13 inhibition with CT7439 synergizes with PARP inhibition in colorectal cancer cell lines. A) RKO and CRC Cell Line 1 colorectal cancer cells were treated with BSJ-4-116 at 50nM for 24 hours and levels of BRCA1 isoforms were measured via qPCR. Error bars indicate SEM. Unpaired t-tests were used to generate p-values. “ns” represents p>0.05.
